# Synthesis, biological evaluation, and molecular docking of novel 1,3,4-substituted-thiadiazole derivatives as potential anticancer agent

**DOI:** 10.1186/s13065-024-01196-1

**Published:** 2024-06-27

**Authors:** Samin A. Shaikh, Satish N. Wakchaure, Shivaji R. Labhade, Raju R. Kale, Rajasekhar R. Alavala, Santosh S. Chobe, Kamlesh S. Jain, Hrishikesh S. Labhade, Dipak D. Bhanushali

**Affiliations:** 1 Department of Chemistry, Savitribai Phule Pune University, Kr. V. N. Naik Shikshan Prasarak Sanstha’s Arts, Commerce and Science College, Canada Corner, Nashik, Maharashtra 422002 India; 2 Department of Synthetic R & D, Delta Finochem Pvt. Ltd., G. No. 350, Wadivarhe, Igatpuri, Nashik, Maharashtra 422403 India; 3 Department of Chemistry, Savitribai Phule Pune University, KTHM College, Nashik, Maharashtra 422002 India; 4 SVKM’s NMIMS, Shobhaben Pratapbhai Patel School of Pharmacy & Technology Management, Vile Parle (W), Mumbai, Maharashtra 400056 India; 5 Department of Chemistry, Savitribai Phule Pune University, M.G.Vs. L. V. H. Arts, Science and Commerce College, Panchavati, Nashik, Maharashtra 422003 India; 6 Friedrich Alexander University Erlangen-Nuremberg (FAU), 91058 Erlangen, Germany

**Keywords:** Anticancer agents, Thiazole-Thiadiazole compounds, Antiproliferative activity, Synthesis optimization, Molecular docking

## Abstract

In an attempt to develop potent anti-cancer agents, a new 1,3,4-substituted-thiadiazole derivatives (**8b-g**), starting from 4-substituted-thiazol-2-chloroacetamides (**4b-g**), were synthesized and evaluated for their cytotoxic effects on multiple human cancer cell lines, including the hepatocellular carcinoma (HEPG-2), human lung carcinoma (A549), human breast carcinoma (MCF-7) and pseudo-normal human embryonic liver (L02) cancer cell lines by an MTT assay. Among all synthesized compounds, compound **8d** showed the potent anti-cancer activities with GI_50_ values of **2.98**, **2.85** and **2.53** μM against MCF-7, A549 and HepG-2 cell lines respectively as compared to standard drug Doxorubicin. Furthermore, molecular modelling studies have spotlighted the anchoring role of 1,3,4-substituted-thiadiazole moiety in bonding and hydrophobic interaction with the key amino acid residues. Therefore, these results can provide promising starting points for further development of best anti-cancer agents.

**Graphical Abstract:**

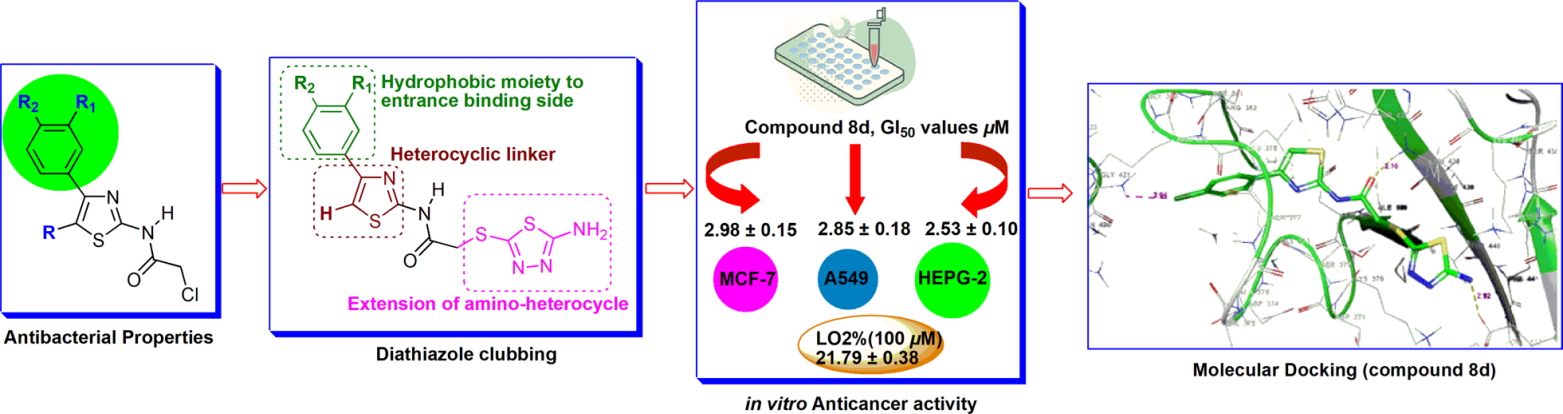

**Supplementary Information:**

The online version contains supplementary material available at 10.1186/s13065-024-01196-1.

## Introduction

Cancer is presently one of the main causes of death worldwide. The International Agency for Research on Cancer (IARC) estimated that globally, 1 in 5 people develop cancer during their lifetime, and 1 in 8 men and 1 in 11 women die from the disease suggesting that more than 50 million people are living within five years of a past cancer diagnosis [1]. In past decades, anticancer research on the design of effective oncology drugs for the application in chemotherapy has improved treatments which led to remarkable results and many drugs have been approved [2, 3]. However, many of the approved drugs still being characterized by high systemic toxicity mainly due to the lack of tumor selectivity and present pharmacokinetic drawbacks, including low water solubility, that negatively affect the drug circulation time and bioavailability which limit its clinical application [4]. The above stated disadvantages of conventional anticancer drugs are the reason why the development of new anticancer drug or improvement in present anticancer drug is still demanding. Therefore, the newer generations of CDK9 inhibitors are already raising as an anticancer therapy and present ongoing research in this direction are helping to develop better, more selective inhibitors.

**Fig. 1:**
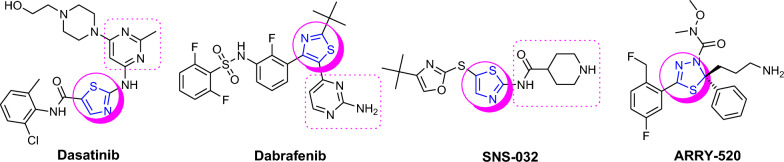
Some examples of thiazole and diathiazole scaffold-based (marked in pink circle) anti-cancer drugs

Thus, the best suitable inhibition strategies are required to be able to distinguish between normal rapidly proliferating cells like the T cells and cancer cells. Thiazole and thiadiazole possess unique properties that make them useful scaffolds in medicinal chemistry [5–10], few examples are shown in Fig. 1. As bioisosteres of pyrimidines, their derivatives can potentially interact with DNA and RNA and can readily cross cell membranes and interact with biological compounds in unique ways. For example, Filanesib (Array-520, Fig. 1) is anti-cancer drug used specifically for multiple myeloma. The best strategy showed when it was combined with bortezomib and dexamethasone had a favorable safety profile. The resistance to Dabrafenib (Fig. 1) and other BRAF inhibitors could be reduced when the Dabrafenib was combined with the MEK inhibitor Trametinib [11]. Recently Noblejas-López et al. had verified that CDK9 PROTAC THAL-SNS-032 showed potent and efficient anti-tumor properties [12]. However, no clinical trials of THAL-SNS-032 (Fig. 1, see structure SNS-032) have been initiated till date.

**Fig. 2:**
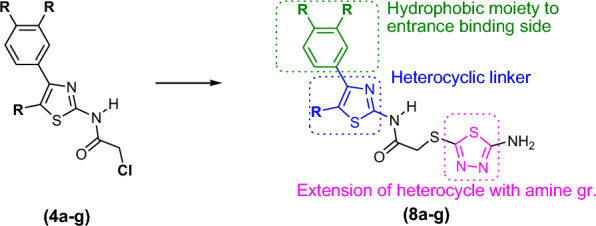
Structural modifications of 4-substituted-thiazol-2-chloroacetamides (**4a-g**) clubbed with 5-amino-1,3,4-thiadiazole-2-thiol (**7**) moiety

Literature data revealed that many research groups used these scaffolds as a basic core for the development of new molecules for anticancer activity. In this study, we have followed the same strategy and proposed to synthesize novel derivatives (**8b-g**) having two basic scaffolds, on one side is thiazole ring and on another side is 5-aminodiathiazole moiety that could mimic Dabrafenib’s side chain (2-aminopyrimidine) to some extent (Fig. 2). Additionally, hydrophobic side chains on these scaffolds could offer the hydrophobic sphere in a molecule which could contribute to enhance the CDK9 selectivity.

## Results and discussion

To confirm the anticancer properties for our new compounds (**8b-g**), first we have referred our previously reported compounds (**4a-g**) [13], and interestingly, the docking studies of them confirmed their potential affinity to selective CDK9/Cyclin T1 (1BLQ), inhibitors (Fig. 4a and Table 2). Based on this results; we proposed the design and synthesis of 1,3,4-substituted-thiadiazole compounds (**8a-g**) as shown in Fig. 2. Although, Gang Yan et al. has reported the compound **8a**, however its anticancer activity is not yet fully explored [14]. The structures of the newly synthesized compounds were elucidated by FT-IR, ^1^H NMR, ^13^C NMR, and LC − MS analysis methods. These compounds (**8a-g**) were screened for in vitro anticancer activity against hepatocellular carcinoma cell lines (HEPG-2), human lung carcinoma cell line (A549) and human breast carcinoma cell (MCF-7) and pseudo-normal human embryonic live cell line (L02) by **MTT** assay. In addition, molecular docking studies were applied to investigate the anti-proliferative effects of these novel compounds.

### Synthetic chemistry

**Scheme 1:**
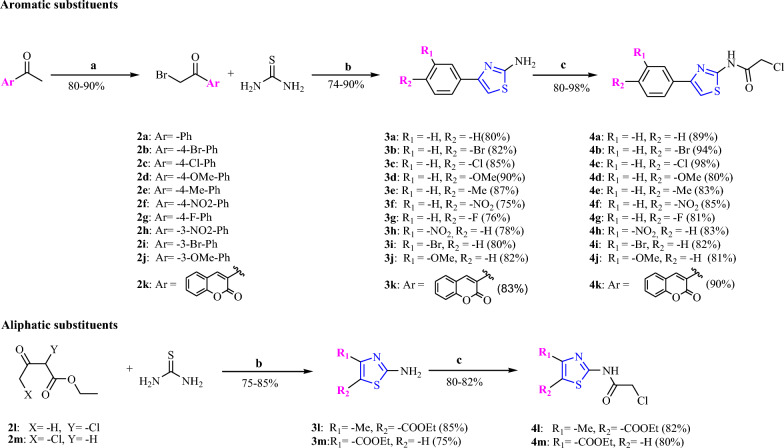
Synthesis of 4-substituted-thiazol-2-chloroacetamide compounds (**4a-m**); Reagents and conditions: **a**. Bromination: Br_2_/AcOH, 0–5 °C, 3 h, (for **2k**: Bromination: Br_2_/CHCl_3_, 55 °C, 4 h); **b** Hantzsch reaction: thiourea, EtOH, reflux, 8h; **c** Amidation: CAC, TEA, DMF, 0–5 °C, 0.5–2.5 h

First we have synthesized 4-substituted-thiazol-2-chloroacetamide compounds (**4a-m**) starting from aromatic ketones and ethyl acetoacetates derivatives in three steps using synthetic methods reported in our previous research articles (Scheme 1) [13] and used them for the synthesis of 1,3,4-substituted-diathiazole derivatives (**8a-g**). The synthetic protocols for (**4a-m**) and (**8a-g**) are given in the Additional file 1 (see Additional file 1 for general experimental procedure).

The intermediates 2-aminothiazoles derivatives (**3a-m**) were prepared by reaction of aromatic ketones and Br_2_ in acetic acid or chloroform followed by Hantzsch reaction of direct condensation of intermediates (**2a-m**) with thiourea in a good to excellent yields. Further amidation reaction using -**CAC** (chloro acetyl chloride) in DMF furnished 4-substituted-thiazol-2-chloroacetamide derivatives (**4a-m**) in excellent yield (Scheme 1). We have followed reported procedure and optimized reaction conditions to obtain these compounds in better yield [13, 15–19].

**Scheme 2:**
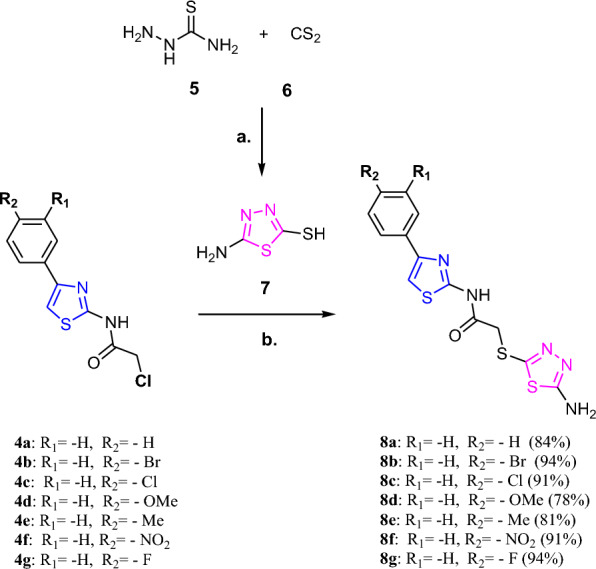
Synthesis of substituted-thiadiazole derivatives (**8a-g**); Reagents and conditions: **a.** NaHCO_3_, EtOH, reflux, 5 h, 90%; **b.** K_2_CO_3_, THF, rt, 0.5–1 h, 78–94%

Our main focus was to synthesize 1,3,4-substituted-thiadiazoles derivatives (**8a-g**). The synthetic strategy has been developed by clubbing of the 4-substituted-thiazol-2-chloroacetamide compounds (**4a-g**) with 5-amino-1,3,4-thiadiazole-2-thiol (**7**) via thial linkage to furnish thiadiazole derivatives (**8a-g**). The optimized reaction condition for (**8a-g**) is depicted in Scheme 2. The excellent yield of these derivatives (**8a-g**) was achieved by substitution reaction of compounds (**4a-g**) with 5-amino-1,3,4-thiadiazole-2-thiol (**7**) in THF at room temperature (Scheme 2, 78–24%). Surprisingly, the substitution reaction worked well for any substituent present on thiazole ring such as -Ph, -4-Br-Ph,-4-Cl-Ph, -4-OMe-Ph, -4-Me-Ph, -4-NO_2_-Ph and -4-F-Ph (Scheme 2, 8a-g).

The **8d** was synthesized with less yield (**8d**:78%), this may be due to the strong positive resonance effect of -OMe group (R_2_) present on the phenyl ring. Especially, when -4-Br-Ph, -4- Cl-Ph, -NO_2_-Ph and -4-F-Ph groups were present on thiazole ring then highest yields observed (Scheme 2, 8b, 8c, 8f and 8 g: 91–94%). This may be due to the negative resonance effect of -NO_2_ group and strong electro-negative atoms such as -Br, -Cl, and -F which made electrophilic substitution reaction better for the 4-substituted-thiazol-2-chloroacetamide compounds **4a** to **4 g**.

### Anti-proliferative activity

Several structural alterations in compounds (**8a-g**) in relation to the substitution at C4 of the thiazole moiety were made to examine whether the nature of the substituent would affect selective toxicity for hepatocellular carcinoma cell lines (HEPG-2), human lung carcinoma cell line (A549) and human breast carcinoma cell line (MCF-7) and pseudo-normal human embryonic liver cell line (L02) by MTT assay. The doxorubicin is used as a standard drug; it is a reasonable standard cytotoxic agent that is a well-known chemotherapy medication. Interestingly, the compounds **4c**, **4j**, **8b**, **8d** and **8f** showed the best anti-proliferative activity with IC_50_ values in the range of **1.82** to **4.07** µM against the MCF-7 cell line. They were also equally potent against A549 and HepG-2 cell lines (GI_50_ values in the range of **2.61** to **18.43** and **2.38** to **5.86** µM respectively) as compared to the doxorubicin (GI_50_ = **3.26** and **3.87** µM respectively, Table 1).

**Table 1:** Structures and in vitro biological activities of compounds (**4a-m**) and (**8a-f**)


	Substituent		Growth inhibition, GI_50_ (*μ*M)^b^			(% inhibition at 100 µM)^b^
Compound^b^	R_1_	R_2_	MCF-7	A549	HepG-2	L02
**4a**	− H	− H	23.76 ± 0.24	18.74 ± 0.41	12.86 ± 0.25	21.63 ± 0.43
**4b**	− H	− Br	18.25 ± 0.39	2.97 ± 0.19	3.62 ± 0.14	17.82 ± 0.51
**4c**	− H	− Cl	2.63 ± 0.14	3.86 ± 0.22	3.92 ± 0.17	18.45 ± 0.48
**4d**	− H	− OMe	26.54 ± 0.32	15.41 ± 0.23	8.37 ± 0.23	12.76 ± 0.22
**4e**	− H	− Me	31.84 ± 0.46	13.67 ± 0.34	7.28 ± 0.16	15.86 ± 0.24
**4f**	− H	− NO_2_	29.43 ± 0.32	19.25 ± 0.32	18.92 ± 0.29	28.16 ± 0.27
**4g**	− H	− F	36.68 ± 0.41	17.54 ± 0.26	22.65 ± 0.46	16.26 ± 0.21
**4h**	− NO_2_	− H	38.15 ± 0.27	28.62 ± 0.47	25.81 ± 0.23	18.43 ± 0.34
**4i**	− Br	− H	33.74 ± 0.32	31.36 ± 0.36	15.96 ± 0.43	17.64 ± 0.38
**4j**	− OME	− H	1.82 ± 0.21	2.61 ± 0.12	2.38 ± 0.24	17.29 ± 0.23
**4k**	Ar = −		26.35 ± 0.28	25.28 ± 0.39	14.26 ± 0.28	22.97 ± 0.35
**4l**	Me	COOEt	37.26 ± 0.37	32.85 ± 0.46	29.83 ± 0.34	26.04 ± 0.47
**4m**	COOEt	H	12.38 ± 0.42	23.68 ± 0.43	4.35 ± 0.16	16.34 ± 0.28
**8a**	− H	− H	34.87 ± 0.31	36.23 ± 0.39	26.75 ± 0.28	22.56 ± 0.42
**8b**	− H	− Br	4.07 ± 0.19	18.43 ± 0.46	5.86 ± 0.24	15.76 ± 0.28
**8c**	− H	− Cl	20.68 ± 0.36	7.86 ± 0.24	4.12 ± 0.18	18.25 ± 0.22
**8d**	− H	− OMe	2.98 ± 0.15	2.85 ± 0.18	2.53 ± 0.10	21.79 ± 0.38
**8e**	− H	− Me	27.99 ± 0.34	29.76 ± 0.37	18.28 ± 0.34	23.76 ± 0.42
**8f**	− H	− NO_2_	3.71 ± 0.26	4.64 ± 0.23	3.46 ± 0.13	20.65 ± 0.37
**doxorubicin**	–	–	4.15 ± 0.18	3.26 ± 0.24	3.87 ± 0.16	82.15 ± 1.26

^a^The cytotoxicity was detected using the **MTT** assay; ^b^The data are expressed as the mean ± SD of three independent experiments

As hypothesized, the compound **4a** has unsubstituted phenyl ring and exhibited poor growth anticancer inhibition properties with GI_50_ values of **23.76, 18.74**, **12.86** and **21.63** µM in all cancer cell lines (Table 1). Substitution at the *-para* position of phenyl ring of **4a** by halogen -bromo (**4b**) resulted moderate growth inhibition properties against MCF-7 cell line (Table 1, GI_50_ of **18.25*** µM*) while it showed potent growth inhibition against A549 and HepG-2 cell lines with GI_50_ values of.

**2.97** and **3.62** µM respectively as compared to the standard doxorubicin (Table 1). Here, the activity was improved, this may be due to the *-para* bromo substituted phenyl ring. Introduction of another substituent by –chloro in *–para* position of phenyl ring of **4a** brought the greatest improvement in the activity of the resulting compound **4b** against MCF-7, A549 and HepG-2 cell lines (GI_50_ value of **2.63**, **3.86** and **3.92** µM respectively) and indicating it was superior in potency against MCF-7 cell line compared to the doxorubicin (Table 1). This potency trend could be explained by the *-para* substituted halogen could have a potential role of drug–target binding affinity in above three cell lines.

In the next stage of biological activity, more compounds with substituted phenyl groups (electron-donating and electron-withdrawing) installed on the C4- and C5-positions of phenyl ring were prepared including substituents -OMe, -Me, -NO_2_ and -F (**4d**, **4e**, **4f, 4 g, 4 h and 4i**) diminished the potency against all four cell lines, (Table 1). Surprisingly, compound **4j** has -methoxy substituent at *-meta* position of phenyl ring exhibited highest potent growth anticancer inhibition properties against MCF-7, A549 and HepG-2 cell lines (Table 1, GI_50_ of **1.82**, **2.61** and **2.38** µM respectively) as compared to the doxorubicin (GI_50_ of **4.15**, **3.26** and **3.87** µM respectively), this is mainly because of electrons donating effect of -methoxy group at –meta position could constructs π electrons interactions and contributing positively for the anti-proliferative activity (Table 1).

To learn the impact of the substituted-diathiazole compounds to potency, we synthesized compounds (**8a-g**) and screened for the anticancer activity. The substitution reaction on **4a** by 5-amino-1,3,4-thiadiazole-2-thiol moiety resulted compound **8a** in moderate reduction of antiproliferative activity as compared to **4a** (Table 1). However, for –*para* substituted chloro compound **8b** significantly increased anticancer inhibition properties against MCF-7 and HepG-2 cell lines (Table 1, GI_50_ value of **4.07** and **5.86** µM respectively) as compared to the compound **4b**. This highlighted 5-amino-thiadiazole moiety and 4-substituted halogen of phenyl ring were playing a major role for the good activity by exploring binding sides to CDK9/Cyclin T1. In this series, compound **8d** represented remarkable potential in MCF-7, A549 and HepG-2 cell lines (Table 1, GI_50_ of **2.98, 2.85**, and **2.53** µM respectively) as compared to doxorubicin. Amazingly, compound **8f** has strong electron-withdrawing substituent *-para* nitro of phenyl ring showed better activity as compared to **4f** in MCF-7, A549 and HepG-2 cell lines with GI_50_ of **3.71**, **4.64** and **3.46** µM respectively and still have comparable activity with doxorubicin (Table 1).

Thus, the compounds **8d** and **8f** have showed promising activity against MCF-7, A549 and HepG-2 cell lines as compared compounds (**4a-i**) and doxorubicin. This is indicating the crucial role of 5-amino-thiadiazole moiety in the potency.

### Annexin V-FITC/PI assay

**Fig. 3:**
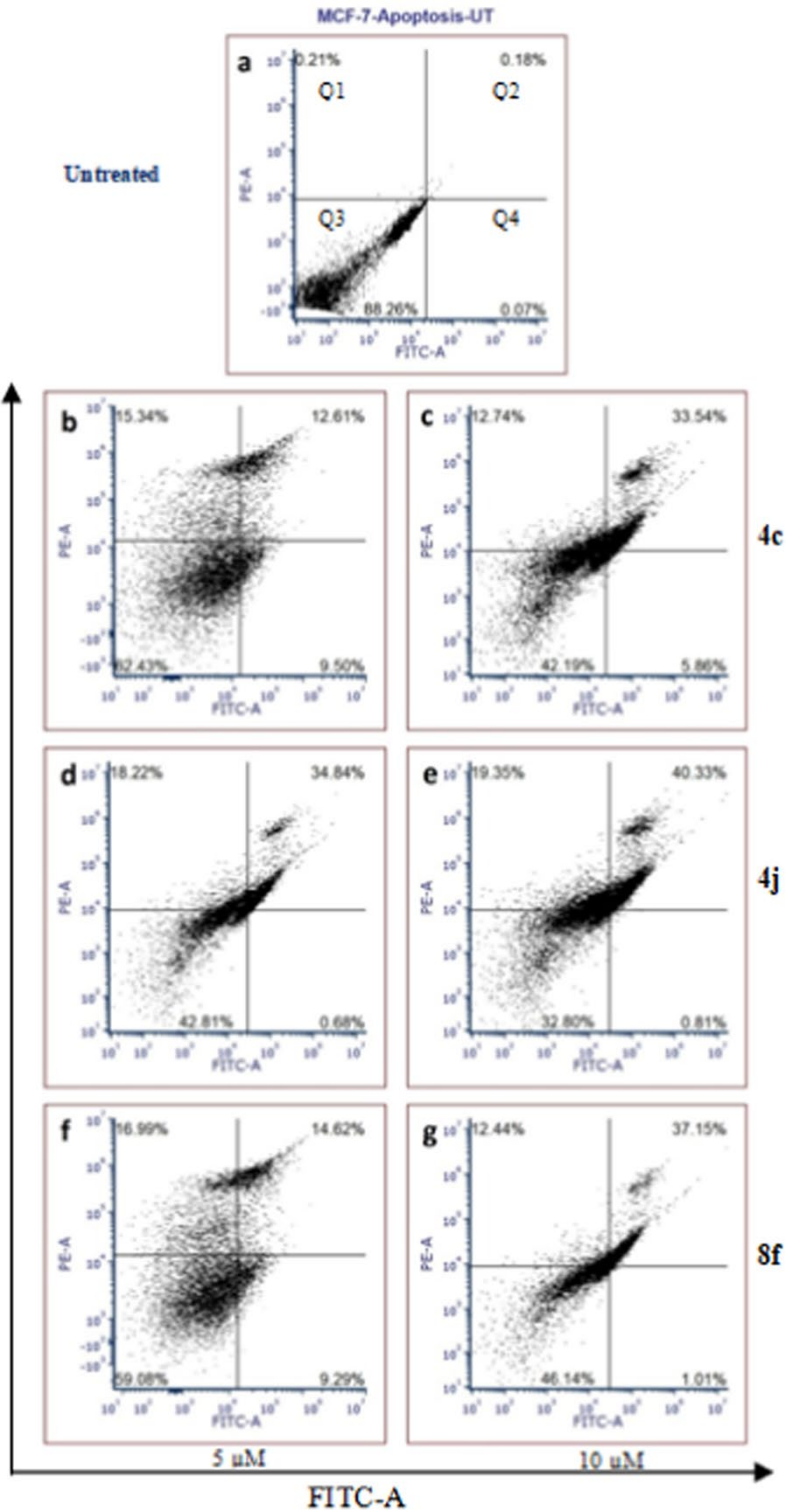
Cell death (apoptosis) analysis of compounds **4c**, **4j** and **8f** on breast carcinoma cell line (MCF-7). The cells were treated with increasing concentrations for 24 h and analyzed by flow cytometry. First quadrant Q1 represents necrotic cell, second quadrant Q2 represents late apoptotic, third quadrant Q3 represents live cells, and forth quadrant Q4 represents early apoptotic cells. The untreated cells have shown 88.26% of live cells, 0.21% death of cells due to necrosis, 0.18% due to the late apoptosis and 0.07% because of the early apoptosis. The experiments were repeated twice and representative data were presented

The early stages of apoptosis were monitored by Annexin V-FITC (apoptotic cell marker) and PI (necrotic cell marker) double staining. The staining method was used according to the Annexin FITC/PI staining kit (Invitrogen™, Thermo Fisher Scientific Inc.). Annexin V- FITC/PI assay was performed to inspect the extent of programmed cell death (Fig. 3, apoptosis) by the compounds **4c**, **4j** and **8f** on breast carcinoma cell line (MCF-7). The cells were treated with increasing concentrations (5–10 μM) for 24 h and analyzed by flow cytometry.

The untreated cells data has shown 88.26% of live cells, 0.21% death of cells due to necrosis, 0.18% due to the late apoptosis and 0.07% because of the early apoptosis (Fig. 3). The compound **4c** has shown 62.43% and 42.19% of live cells at 5 and 10 μM concentration respectively. Whereas the necrosis is prominent cause of the cell growth inhibition at 5 μM and at 10 μM, the late apoptosis is the major reason for the cell death. Compound **4j** has reduced the viable cell count to the 42.81% at 5 μM and at 10 μM it was 32.80% only. The cell death was majorly occurred due to the late apoptosis with 34.84% and 40.33% at the tested concentrations. Compound **4j** has showed considerable reduction in the viable cell count as compared to **4c***.* This may be due to the electron-donating substituent (-methoxy) present on the phenyl ring at *–meta* position of **4j** which have much better hydrophilic interactions due to availability of electrons. Whereas in compound **4c,** the -chloro substituent present on phenyl ring at *–para* position leading minimum reduction of cell counts (Fig. 3). Interestingly, compound **8f** has shown 59.08% and 46.14% of live cells at the tested concentrations. It has shown, the cell death of 16.99 and 14.62% at 5 μM due to necrosis and late apoptosis respectively and at 10 μM, the percentage of death was 12.44% and 37.15%. Thus, this underlined the role of substituted-diathiazole moiety in apoptosis.

### Molecular modeling studies

The target prediction was done by using the Swiss Target Prediction and Pass Online tools. Based on the scores and ratio data from the two servers, potential anti-cancer targets like Cyclin-dependent kinase 9 (CDK9)/Cyclin T1–3BLQ signal transducer and activator of transcription 3 (STAT3) were selected [20, 21]. In addition, Sitemap tool of Glide was used for predicting the binding pockets and one of the predicted five was selected for docking studies based on the score and volume. Glide tool of the Maestro (Schrödinger Release 2023-2, New York, USA) was used for the exploring molecular docking studies. [22–25]

**Fig. 4:**
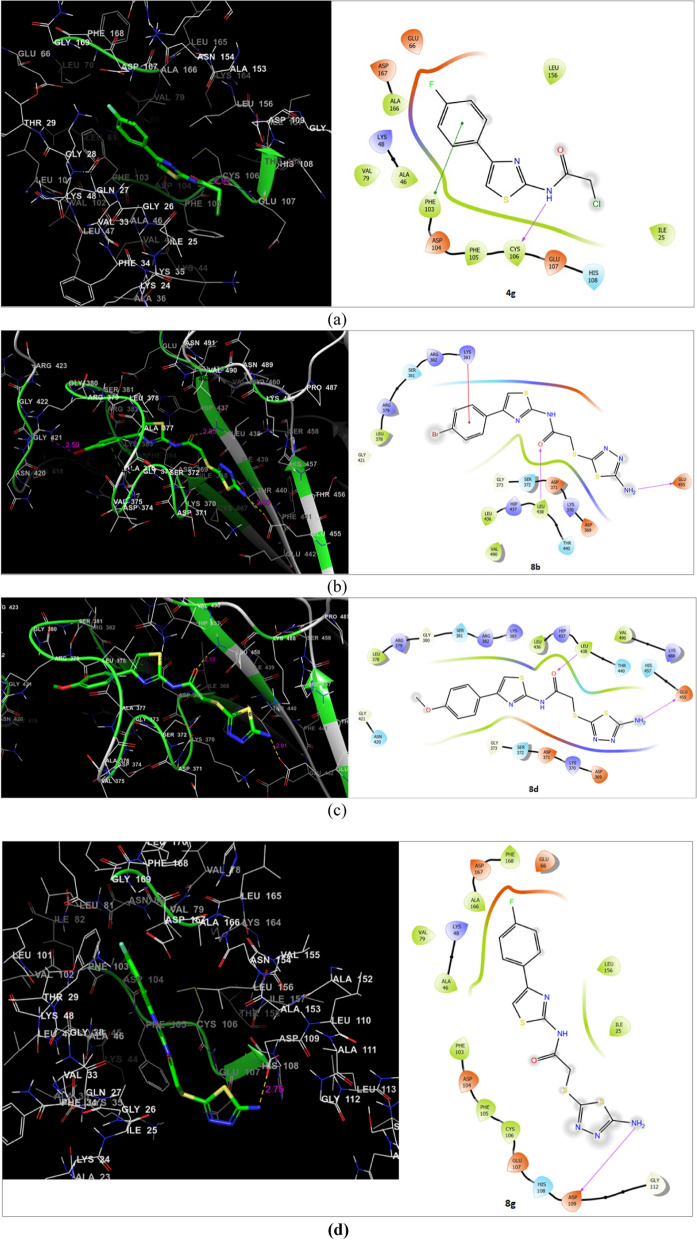
Binding modes of compounds **4g**, **8b**, **8d** and **8g** (from the top of the page figure **a**, **b**, **c** and **d**) with CDK9/Cyclin T1 and an important residues and interaction distances for key residues. CDK9/Cyclin T1 is shown in green color. The figure was prepared using Glide tool of the Maestro (Schrödinger, LLC, New York, USA)

The docking studies of investigated compounds (**4a-m**) displayed XP GScores between − 7.777 and − 3.826 kcal/mol against CDK9/Cyclin T1 (1BLQ), providing the insights about their binding mode in the enzyme's binding cavity. Whereas verifying their potential affinity for the enzyme's binding cavity as compared to standard drug Flavopiridol as shown in Table 2 (Fig. 4). We have chosen reference drug Flavopiridol because it is an Orphan drug used for the treatment of acute myeloid leukemia as well as the treatment of arthritis. Flavopiridol is a known inhibitor of CDK9 and it was used as a standard inhibitor for comparing the docking results. STX-0119 is a co- crystalized ligand in STAT3 protein, it was used for validating the docking protocol and to compare the docking score and energy with the designed compounds. As per the data obtained from the experimentation, some of the designed molecules were found to possess better docking score with the studied protein molecules. Especially, compound **4 g** was showed highest docking score – 7.777 kcal/mol against CDK9/Cyclin T1–1BLQ (Table 2) and found to interact with the binding site residues Cys106 (2.05, 2.66 Å) with H-bonding interactions and π-π stacking with Phe103 (Fig. 4a). As hypothesized, substitution of phenyl derivatives at C4 position of the thiazole ring afforded moderate to potent and relatively selective CDK9 inhibitors and other side of 5-amino-thiadiazole moiety could offers free -NH_2_ and -N groups as a key hydrogen bond donor and acceptor to interact with the backbone carbonyl and the -NH groups of the binding pockets respectively and possibly exhibited potential anticancer properties. Therefore, in this study we designed and synthesized six new 1,3,4-substituted-thiadiazole compounds (**8b-g**) and performed the docking studies for their potential anticancer properties. [12–14]

**Table 2:** Docking score and energy of the tested compounds^a^

Compounds	CDK9/Cyclin T1–3BLQ		STAT3-1BG1	
	Docking score	Glide emodel	Docking score	Glide emodel
4a	− 7.734	− 45.358	− 4.842	− 37.574
4b	− 4.532	− 45.126	− 4.297	− 36.739
4c	− 7.183	− 43.123	− 5.225	− 39.644
4d	− 7.464	− 46.86	− 5.239	− 38.121
4e	− 6.376	− 45.919	− 4.523	− 35.675
4f	− 6.323	− 46.953	− 5.269	− 37.953
4g	− 7.777	− 44.228	− 4.985	− 39.019
4h	− 7.254	− 49.916	− 4.784	− 42.581
4i	− 7.283	− 47.625	− 4.408	− 38.370
4j	− 6.075	− 51.949	− 5.040	− 37.895
4k	− 4.98	− 52.264	− 3.374	− 34.216
4l	− 3.826	− 47.903	− 3.686	− 30.540
4m	− 4.508	− 49.863	− 4.901	− 40.403
8a	− 6.479	− 60.016	− 3.662	− 49.163
8b	− 6.133	− 64.789	− 5.635	− 62.694
8c	− 7.451	− 61.403	− 5.886	− 62.646
8d	− 7.049	− 65.300	− 5.804	− 63.304
8e	− 5.369	− 59.562	− 4.543	− 53.690
8f	− 5.702	− 70.403	− 5.121	− 59.816
8g	− 7.324	− 62.143	− 5.341	− 62.389
STX− 0119	− 6.187	− 61.656	− 4.242	− 60.276
(CPB) (Flavopiridol)	− 6.238	− 53.629	–	–

^a^Calculated using Graph Pad Prism program (Graph Pad software Inc, CA)

In the first series of **4a to 4 m** compounds, as discussed above compound **4 g** has highest GScores (Table 2, − 7.777 kcal/mol), however experimental data revealed moderate growth in anticancer inhibition properties (Table 1). The compound **4c** has also showed good GScores (Table 2, − 7.183 kcal/mol) and exhibited the strong binding interactions with the acidic residues of CDK9/Cyclin T1 and also the experimental data of anti-proliferative activity showed potent anticancer inhibition properties against MCF-7, A549 and HepG-2 cell lines (Table 1, 4c). Therefore, experimental data are in good agreement with docking data of compound **4c**. Remarkably, the compound **4j** showed highest potent growth anticancer inhibition properties experimentally as compared to all other compounds and the docking score for **4j** against CDK9/Cyclin T1–1BLQ (Table 1, docking score = -6.075 kcal/mol and glide emodel = -51.949 kcal/mol) were comparable with the docking score of STX-0119 and CPB as shown in Table 2. This may be because of **4j** has -methoxy substituent at *–meta* position of phenyl ring and it was moderately water soluble and has showed high GI absorption value with fulfilling Lipinski rule of five. However same isomeric compound **4d** which has –methoxy substituent at *–para* position of phenyl ring and showed good dockings core (Table 2, − 7.464 kcal/mol) and found to be inactive practically (Tables 3 and 4). Similarly, for compounds **4e** and **4f** which showed acceptable docking score (Table 2, GScores − 6.376 and − 6.323 and kcal/mol) but experimental data indicating that they showed moderate anti-proliferative activity (Table 1, 4e and 4f). So in this series it was observed that *-para* substituted halogen and *-meta* substituted strong electron donating groups determined an improvement in the potency as compared to other compounds.

**Table 3:** ADME prediction of the designed compounds (**4a-m** and **8a-g**)^a^

Compunds	MW	RB	HBA	HBD	MR	TPSA	CLog P	Log S	ESOL Class
4a	252.72	4	2	1	66.66	70.23	2.48	− 3.38	Sol
4b	331.62	4	2	1	74.36	70.23	3.11	− 4.28	Mod. Sol
4c	287.17	4	2	1	71.67	70.23	3.02	− 3.96	Sol
4d	282.75	5	3	1	73.15	79.46	2.47	− 3.43	Sol
4e	266.75	4	2	1	71.62	70.23	2.82	− 3.67	Sol
4f	297.72	5	4	1	75.48	116.05	1.65	− 3.41	Sol
4g	270.71	4	3	1	66.62	70.23	2.8	− 3.53	Sol
4h	297.72	5	4	1	75.48	116.05	1.62	− 3.41	Sol
4i	331.62	4	2	1	74.36	70.23	3.11	− 4.28	Mod. Sol
4j	282.75	5	3	1	73.15	79.46	2.5	− 3.43	Sol
4k	320.75	4	4	1	82.7	100.44	2.64	− 3.82	Sol
4l	262.71	6	4	1	62.28	96.53	1.77	− 2.59	Sol
4m	262.71	7	4	1	61.89	96.53	1.42	− 2.04	Sol
8a	349.45	6	4	2	90.98	175.57	2.32	− 3.91	Sol
8b	428.35	6	4	2	98.68	175.57	3.05	− 4.81	Mod. Sol
8c	383.9	6	4	2	95.99	175.57	2.94	− 4.49	Mod. Sol
8d	379.48	7	5	2	97.47	184.8	2.33	− 3.96	Sol
8e	363.48	6	4	2	95.94	175.57	2.66	− 4.2	Mod. Sol
8f	394.45	7	6	2	99.8	221.39	1.63	− 3.95	Sol
8g	367.44	6	5	2	90.93	175.57	2.72	− 4.06	Mod. Sol

^a^MW-Molecular Weight; RB-Number of rotatable bonds; HBA Number of H-Bond acceptors; HBD-Number of H-Bond donors; MR-Molar refractivity; TPSA-Total polar surface area; CLog P-Consensus Log P

**Table 4:** ADME prediction of the designed compounds (**4a-m** and **8a-g**)

Compunds	GI absorption	BBB permeant	Pgp substrate	CYP isoform inhibitor					log Kp (cm/s)
				**1A2**	**2C19**	**2C9**	**2D6**	**3A4**	
**4a**	High	Yes	No	Yes	Yes	No	No	No	− 5.89
**4b**	High	Yes	No	Yes	Yes	Yes	No	No	− 5.88
**4c**	High	Yes	No	Yes	Yes	Yes	No	No	− 5.66
**4d**	High	No	No	Yes	Yes	Yes	No	No	− 6.09
**4e**	High	Yes	No	Yes	Yes	Yes	No	No	− 5.72
**4f**	High	No	No	Yes	Yes	No	No	No	− 6.28
**4g**	High	Yes	No	Yes	Yes	No	No	No	− 5.93
**4h**	High	No	No	Yes	Yes	No	No	No	− 6.28
**4i**	High	Yes	No	Yes	Yes	Yes	No	No	− 5.88
**4j**	High	No	No	Yes	Yes	Yes	No	No	− 6.09
**4k**	High	No	No	Yes	Yes	Yes	No	No	− 6.31
**4l**	High	No	No	Yes	No	No	No	No	− 6.45
**4m**	High	No	No	No	No	No	No	No	− 6.99
**8a**	Low	No	No	Yes	Yes	Yes	No	Yes	− 6.45
**8b**	Low	No	No	Yes	Yes	Yes	No	Yes	− 6.44
**8c**	Low	No	No	Yes	Yes	Yes	No	Yes	− 6.21
**8d**	Low	No	No	No	Yes	Yes	No	Yes	− 6.66
**8e**	Low	No	No	Yes	Yes	Yes	No	Yes	− 6.27
**8f**	Low	No	No	No	Yes	No	No	Yes	− 6.85
**8g**	Low	No	No	Yes	Yes	Yes	No	Yes	− 6.49

More interestingly, when compounds (**4a-g**) were extended by introducing amino-diathiazole moiety resulted highly potent compounds **8b**, **8c**, **8d**, **8f** and **8 g** with highest docking score against CDK9/Cyclin T1–1BLQ inhibitor (Table 2, − 6.133, − 7.451, − 7.049, − 5.702 and − 7.324 kcal/mol respectively). However, the experimental data revealed that compound **8c** showed moderate anticancer inhibition properties while compounds **8b**, **8d** and **8f** showed potent anticancer inhibition properties (Table 1). The compound **8b** has formed hydrophobic interaction with Lys and strong Hydrogen bonding with Leu and Glu amino acids residues (Fig. 4b). The compound **8d** has **-**methoxy substituent at –*para* position of phenyl ring formed of strong Hydrogen bonding with Cys106 (2.82 Å and 2.29 Å) and Arg382(2.97 Å) as shown in Fig. 4c. Similarly, compound **8f** has formed interactions with Glu107 (2.77 Å), Cys106 (2.24 Å), Phe103 (2.3 Å), Asp167 (2.19 Å) and Glu66 (2.51 Å) by strong H-bond and ionic interaction with Lys48 as shown in Fig. 4d. Additionally, all compound **8b**, **8d** and **8f** have shown good pharmacokinetic properties as predicted by ADME (Tables 3 and 4).

In the drug likeness prediction and the pharmacokinetic property (ADME) calculations, it was found that all the compounds have molecular weight well below 500 Daltons and they may not face any difficulty to pass the biological membranes when given through oral route (Table 3). The same has been supported by the CLogP, where the compound have shown optimum partition coefficient, between 1.42 to 3.11. Whereas, all the compounds were having a minimum of 4 rotatable bonds required to undergo conformational changes during the binding to a protein target. The Hydrogen bond acceptors and donors were well within the accepted limits to meet the Lipinski rule of five. In addition to that the compounds were found to have high GI absorption and P-gp non-substrate nature (Table 4) makes them a viable choice to take them to next stage of exploration.

Thus, by experimental data, compounds **4j**, **8b**, **8d** and **8f** were found to be the potent anticancer agent. By the interaction energy, it was observed that these compounds have -bromo, -methoxy and -nitro substituent’s at -*para* position of phenyl ring of substituted-diathiazole compounds, which strongly assist the molecules to form potential bonds with the residues of the binding sites. It was proved that the docking calculations and the experimental data are close in proximity.

## Conclusion

A series of novel 1,3,4-substituted-thiadiazole compounds (**8b-g**) designed and synthesized from 4-substituted-thiazol-2-chloroacetamides (**4b-g**) and fully characterized by spectrometric analysis methods (FT − IR, ^1^H NMR, ^13^C NMR, and mass). All synthesized compounds (**4a-m**) and (**8a-f**) exhibited selective anti-proliferative activity. Compounds **4j**, **8d** and **8f** demonstrated promising anticancer activity against MCF-7, A549, HepG-2 and L02 all cancer cell lines. Especially compounds **4j** and **8d** exhibited the most potent anticancer inhibition properties with GI_50_ values of 1.82, 2.61, 2.38 μM and 2.98, 2.85, 2.53 μM against MCF-7, A549 and HepG-2 cell lines respectively as compared to Doxorubicin. Further molecular docking was revealed the biological activity. Thus, these compounds can provide promising starting points for further development of best anti-cancer agents.

### Supplementary Information

**Additional file 1. **Additional figures and sections.

## Data Availability

All data generated or analyzed during this study are included in this published article [and additionally its supplementary information file includes materials and methods, general experimental procedure, NMR data, NMR spectra, mass spectra and IR spectra].
