# Supplementary material for: Synthesis, biological evaluation, and molecular docking of novel 1,3,4-substituted-thiadiazole derivatives as potential anticancer agent
Source: BMC Chem. 2024 Jun 27;18(1):119. doi: 10.1186/s13065-024-01196-1 (PMC11210122; doi:10.1186/s13065-024-01196-1)

**Additional file 1**

**Synthesis, Biological Evaluation, and Molecular Docking of Novel 1,3,4-Substituted-thiadiazole Derivatives as Potential Anticancer Agent**

Samin A. Shaikh^1^*, Satish N. Wakchaure^2^*, Shivaji R. Labhade^3^, Raju R. Kale^3^, Rajasekhar R. Alavala^4^, Santosh S. Chobe^5^, Kamlesh S. Jain^1^, Hrishikesh S. Labhade^1^ and Dipak D. Bhanushali^1^

1. Department of Chemistry, Savitribai Phule Pune University, Kr. V. N. Naik Shikshan Prasarak Sanstha's Arts, Commerce and Science College, Canada Corner, Nashik-422002, MS, India
2. Department of Synthetic R & D, Delta Finochem Pvt. Ltd., G. No. 350, Wadivarhe, Igatpuri, Nashik- 422403, MS, India. Email: satish_nclpune@yahoo.co.in
3. Department of Chemistry, Savitribai Phule Pune University, KTHM College, Nashik-422002, MS, India.
4. SVKM’s NMIMS, Shobhaben Pratapbhai Patel School of Pharmacy & Technology Management, Vile Parle (W), Mumbai- 400056, MS, India.
5. Department of Chemistry, Savitribai Phule Pune University, M.G.Vs. L. V. H. Arts, Science and Commerce College, Panchavati, Nashik-422003, MS, India.

**Corresponding Authors**

1. Dr. Satish N. Wakchaure, Phone No. +91-9016242196, Email. satish_nclpune@yahoo.co.in
2. Samin A. Shaikh, Phone No. +91-7066329706, Email. saminshaikh80@gmail.com

| **Sr. No.** | **Table of Contents** | **Page No.** |
| --- | --- | --- |
| 1. | Synthesis: General Experimental Procedures for (**4a-m**) and (**8a-g**) | S2 |
| 2. | Spectral Data of representative compounds (**8a-g**) | S2 |
| 3. | Materials and Methods | S3 |
| 4. | NMR spectra of representative compounds (**8a-g**) | S5 |
| 5. | Mass Spectra for (**8a-g**) | S12 |
| 6. | IR Spectra for (**8a-g**) | S16 |

1. Synthesis

General experimental procedures

The alpha halo ketone derivatives (2a-m), 2-aminothiazole derivatives (3a-m) and 4-substituted-thiazol-2-chloroacetamide derivatives (4a-m) are known compounds and has been synthesized using new optimized reaction conditions. The purity is acceptable by ^1^H NMR data.

General Procedures for the synthesizing 2-bromo-1-phenylethanone derivatives (2a-j)

A four-necked, round-bottomed flask was charged with acetophenone derivative 1a-j (10.0 mmols) and glacial acetic acid (20 ml). The flask was immersed in an ice salt mixture (0 – 5 °C), and bromine (10.0mmoles) was added from the pressure equalizing funnel at such a rate that the temperature of the reaction mixture did not exceed 5°C. After completion of the addition, it was stirred at RT for 2 – 3 hours. The suspension was poured onto crushed ice. The precipitate was filtered, repeatedly washed with water, and dried at room temperature (TLC mobile phase. chloroform).

Experimental Procedure for 3-(2-bromoacetyl)-2H-chromen-2-one (2k)

3-Acetylcoumarin (1k, 10.0 mmoles) was dissolved in alcohol-free chloroform (20 mL), and a solution of bromine (10.0 mmoles) in chloroform (5 ml) was added dropwise from a dropping funnel. The mixture was stirred at room temperature for 15 minutes and then heated to 40 – 45 °C for 20 minutes on a water bath to expel most of the hydrogen bromide. The solid obtained was washed by ether and purified by recrystallization from glacial acetic acid (TLC mobile phase. n-hexane: ethyl acetate = 8:2).

General Procedures for the synthesizing thiazol-2-amino derivatives (3a-m)

The mixture of 2-bromo-1-phenylethanone derivatives 2a-j or 2l or 2m or 3-(2-bromoacetyl)-2H-chromen-2-one (2k) (10.0 mmoles), thiourea (11.0 mmoles) and ethanol (10 ml) was refluxed in four necked round-bottomed flasks at 78 °C. The reaction was completed within 1 – 2 hours. The reaction mixture was poured into the crushed ice. The products are filtered and washed with water and ethanol. The products were recrystallized by using ethanol as a solvent (TLC mobile phase. n-hexane: ethyl acetate = 6:4).

General Procedures for the acylation of thiazol-2-amino derivatives (4a-m)

The mixture of thiazol-2-amino derivatives (3a-m, 1.0 mmoles) and triethylamine (1.3 mmoles) was taken in a round-bottom flask. Reaction mass was stirred at room temperature for 30 minutes. To this reaction, the chloroacetyl chloride (CAC, 2.2 mmoles) was added at 0 – 5 °C. The reaction was completed within 1 – 4 hours. The reaction mixture is washed with a brine solution and then water. The solution was neutralised by a 1:1 HCl solution. The products were filtered and washed with water (TLC mobile phase. n-hexane: ethyl acetate = 6:4).

General procedures for the synthesizing 4-substituted-2-amino-thiadiazole-thio-thiazole-acetamide compounds (8a-g). A solution of 5-amino-1,3,4-thiadiazole-2-thiol (7, 1.0 mmoles) and potassium carbonate (1.1 mmoles) in THF (10 ml) was stirred at ambient temperature for 30 minutes. Subsequently, a solution of acylated 2-aminothiazole derivative: 4a-m (1.0 mmole) in THF (5 ml) was added drop-wise by dropping a funnel into the reaction mass. Reaction mass was stirred at room temperature and confirmed the completion of the reaction by TLC. After the reaction was completed, the reaction mixture was poured into crushed ice. The product was filtered, washed with brine solution, and recrystallized from ethyl acetate to give a pure compound (TLC mobile phase. Toluene: ethyl acetate: aq. Formaldehyde = 5:4:1). The purity is acceptable by ^1^H NMR data.

**2. Spectral Data for Representative Compounds (8a-g).**

**2-(5-amino-1,3,4-thiadiazol-2-ylthio)-N-(4-phenylthiazol-2-yl) acetamide (8a):**

FT.IR (in cm^-1^):620.71 (C-H stretching), 1178.75 (C-N stretching), 1335.61 (-C-O), 1510.94 (aromatic), 1616.37 (amide O=C-NH), 3475.71 (amine N-H). ^1^H-NMR (500 MHz, DMSO-d_6_) *δ* 4.11 (s, 2H), 7.37 – 7.29 (m, 2H), 7.44 (m, 1H), 7.56 (s, 1H), 7.66 (s, 2H), 7.90 (m, 2H), 12.53 (s, 1H). ^13^C-NMR (126 MHz, DMSO-d_6_) *δ* 37.19, 108.25, 125.55, 127.74 (strong), 128.48, 128.64 (strong), 134.06, 148.68, 148.84, 166.39, 170.01. Mol. Formula. C_13_H_11_N_5_OS_3_, Mol. Wt.: 349.45. MS: Exact Mass: 349.4450; Observed Mass: 349.7523. Calculated: Elem. Anal: C, 44.68%; H, 3.17%; N, 20.04%; O, 4.58%; S, 27.53%

**2-(5-amino-1,3,4-thiadiazol-2-ylthio)-N-(4-(4-bromophenyl) thiazol-2-yl) acetamide (8b):**

FT.IR (in cm^-1^):620.73 (C-H stretching), 1177.97 (C-N stretching), 1383.05 (-C-O), 1516.65 (aromatic), 1617.57 (amide O=C-NH), 3470.61(amine N-H). ^1^H-NMR (500 MHz, DMSO-d_6_) *δ* 4.20 (s, 2H), 7.42 (s, 2H), 7.72 (d, J = 8.5 Hz, 2H), 7.82 (s, 1H), 7.94 (d, J = 8.5 Hz, 2H), 12.65 (s, 1H). ^13^C-NMR (126 MHz, DMSO-d_6_) *δ* 37.17, 109.08, 120.81, 127.58 (strong), 131.58 (strong), 133.27, 147.65, 148.66, 157.71, 166.48, 170.01. Mol. Formula. C_13_H_10_BrN_5_OS_3,_ MS: Exact Mass: 428.3410; Observed Mass: 427.9331 (major), 429.9310 (major). Calculated: Elem. Anal: C, 36.45%; H, 2.35%; Br, 18.65%; N, 16.35%; O, 3.74%; S, 22.46

**2-(5-amino-1,3,4-thiadiazol-2-ylthio)-N-(4-(4-chlorophenyl) thiazol-2-yl) acetamide (8c):**

FT.IR (in cm^-1^):620.54 (C-H stretching), 744.74 (para di substituted), 1181.57(C-N stretching), 1385.57 (-C-O), 1502.23 (aromatic), 1617.46 (amide O=C-NH), 3476.72 (amine N-H). ^1^H-NMR (500 MHz, DMSO-d_6_) *δ* 4.11 (s, 2H), 7.33 (s, 2H), 7.50 (d, J = 6.0 Hz, 2H), 7.72 (s, 1H), 7.91 (d, J = 6.0 Hz, 2H), 12.55 (s, 1H). ^13^C-NMR (126 MHz, DMSO-d_6_) *δ* 37.17, 109.00, 127.27 (strong), 128.67 (strong), 132.21, 132.93, 147.61, 148.56, 157.71, 166.47, 170.00. Mol. Formula. C_13_H_10_ClN_5_OS_3,_ MS: Exact Mass: 383.8870; Observed Mass: 380.0336 (major), 381.0355 (minor). Calculated: Elem. Anal: C, 40.67%; H, 2.63%; Cl, 9.23%; N, 18.24%; O, 4.17%; S, 25.06%

**2-(5-amino-1,3,4-thiadiazol-2-ylthio)-N-(4-(4-methoxyphenyl) thiazol-2-yl) acetamide (8d):**

FT.IR (in cm^-1^):619.89 (C-H stretching), 732.11 (para di substituted), 814.13 (para di substituted), 1041.71(C-N stretching), 1384.58 (-C-O), 1502.29 (aromatic), 1616.32 (amide O=C-NH), 3476.55 (amine N-H). ^1^H-NMR (500 MHz, DMSO-d_6_) *δ* 3.77 (s, 3H), 3.86 (s, 2H), 6.81 (s, 1H), 6.92 (d, *J* = 8.8 Hz, 2H), 6.99 (s, 2H), 7.72 (d, *J* = 8.8 Hz, 2H), 12.45 (s, 1H). ^13^C -NMR (126 MHz, DMSO-d_6_) *δ* 21.14, 66.98, 106.34,125.35 (strong), 128.88 (strong), 132.51, 136.41, 148.57, 150.79, 164.72, 167.61, 169.35. Mol. Formula. C_14_H_13_N_5_O_2_S_3,_ MS: Exact Mass: 379.4710; Observed Mass: 380.00. Calculated: Elem. Anal: C, 44.31%; H, 3.45%; N, 18.46%; O, 8.43%; S, 25.35%.

**2-(5-amino-1,3,4-thiadiazol-2-ylthio)-N-(4-p-tolylthiazol-2-yl) acetamide(8e):**

FT.IR (in cm^-1^):620.53 (C-H stretching), 736.47 (para di substituted), 825.92 (para di substituted), 1052.26 (C-N stretching), 1384.16 (-C-O), 1518.44 (aromatic), 1617.58 (amide O=C-NH), 3475.42 (amine N-H). ^1^H-NMR (500 MHz, DMSO-d_6_) *δ* 2.32 (s, 3H), 4.02 (s, 2H), 7.20 (d, *J* = 7.9 Hz, 2H), 7.28 (s, 2H), 7.34 (s, 1H), 7.42 (s, 1H), 7.77 (d, *J* = 8.1 Hz, 2H). ^13^C-NMR (126 MHz, DMSO-d_6_) δ 37.22, 40.32, 108.04, 115.43, 115.60(strong), 127.57, 127.63(strong), 130.72, 147.86, 157.66, 166.45, 170.06. Mol. Formula. C_14_H_13_N_5_OS_3,_ LCMS: Exact Mass: 363.4720; Observed Mass: 363.0960. Calculated: Elem. Anal: C, 46.26%; H, 3.60%; N, 19.27%; O, 4.40%; S, 26.46%.

**2-(5-amino-1,3,4-thiadiazol-2-ylthio)-N-(4-(4-nitrophenyl) thiazol-2-yl) acetamide(8f):**

FT.IR (in cm^-1^):620.53 (C-H stretching), 736.47 (para di substituted), 825.92 (para di substituted), 1052.26 (C-N stretching), 1384.16 (-C-O), 1518.44 (aromatic), 1617.58, 3475.42 (amine N-H). ^1^H-NMR (500 MHz, DMSO-d_6_) *δ* 4.35 (s, 2H)), 7.97 (s, 1H), 8.08 (d, *J* = 9.0 Hz, 2H), 8.23 (d, *J* = 9.0 Hz, 2H), 12.67 (bs, 3H), ^13^C-NMR (126 MHz, DMSO-d6) δ 57.41, 101.01, 115.01 (strong), 127.64 (strong), 129.60, 131.73, 135.01, 148.65, 160.42, 162.57, 168.15. Mol. Formula. C_13_H_10_N_6_O_3_S_3,_ MS: Exact Mass: 394.4420; Observed Mass: 395.00. Calculated: Elem. Anal: C, 39.58%; H, 2.56%; N, 21.31%; O, 12.17%; S, 24.39%.

**2-(5-amino-1,3,4-thiadiazol-2-ylthio)-N-(4-(4-fluorophenyl) thiazol-2-yl) acetamide (8g):**

FT.IR (KBr disc) (cm^-1^):621.12 (C-H stretching), 754.81(para di substituted), 1055.32 (C-N stretching), 1381.31 (-C-O), 1514.22 (aromatic), 1617.04 (amide O=C-NH), 3414.20(amine N-H). ^1^H-NMR (500 MHz, DMSO-d_6_) *δ* 3.47 (s, 2H), 6.98 (s, 1H), 7.06 (s, 2H), 7.95 -7.19 (m, 4H), 13.17 (s, 1H). ^13^C-NMR (126 MHz, DMSO-d_6_) *δ* 40.01, 101.06, 115.03, 115.20 (strong), 127.29, 127.35 (strong), 131.42, 148.67, 160.29, 162.23, 168.17. Mol. Formula. C_13_H_10_FN_5_OS_3,_ MS: Exact Mass: 367.4354; Observed Mass: 367.0978 (major). Calculated: Elem. Anal: C, 42.49%; H, 2.74%; F, 5.17%; N, 19.06%; O, 4.35%; S, 26.18%

3. Materials and methods

General consideration

All reagents and solvents were commercially available in analytical grade and used as received. Unless otherwise specified, yields refer to chromatographically and spectroscopy (^1^H NMR) homogeneous materials. The solvents that were used were filtered and dried using standard techniques. A mass spectrum (MS) of each compound was obtained with a FTICR-MS (Ionspec 7.0 T) spectrometer, Punjab University. Elemental C, H, N, and S analysis was completed by the K-factor calibration method at Saif, Punjab University. Thermo Scientific (FLASH 2000) C, H, N Elemental Analyser is a microprocessor-based instrument that determines the CHN Analyzer percentages of C, H, and N with an accuracy of 0.3%. The results are given in the form of a percentage of each element analyzed. LC-MS mass was analysed by Micromass Q-Tof micro mass spectrometer, Punjab University. Fourier-transform infrared spectroscopy (FT-IR) (KBr disc) (cm-1) were recorded at the Laboratory, Punjab University Chandigarh. The ^1^H and ^13^C NMR spectra were recorded in CDCl_3_ or DMSO-d_6_ solutions on a Bruker a Vance Neo 500 MHz NMR Spectrometer (Saif, Punjab University) spectrometer. Chemical shifts are reported in parts per million (ppm) relative to CDCl_3_ (7.27 ppm) for ^1^H NMR data and CDCl_3_ (77.0 ppm) for ^13^C NMR data or the peak of DMSO-d6, defined at = 2.50 (^1^H NMR) or = 39.5 (^13^C NMR). The following abbreviations were used to explain multiplicities: s = singlet, d = doublet, t = triplet, q = quartet, m = multiplet, tt = double triplet and br = broad.

**Analytical Methods**

**Methods for *in vitro* anti-proliferative assay [Cell lines]:** In the current investigation, hepatocellular carcinoma cell lines (HEPG-2), human lung carcinoma (A549) and human breast carcinoma cell line (MCF-7) and pseudo-normal human embryonic liver cellline (L02) were used for antiproliferative activity, the cell lines were obtained from NCL, Pune. The growth medium used is the DMEM (Dulbecco's modified Eagle's growth medium) having 10% FBS (fetal bovine serum), 10 *µ*g/ml of insulin (Sigma) and 1% penicillin/streptomycin. The used reagents and chemicals were purchased from Sigma or Invitrogen. 100 *µ*l of each compound was added to cells with density of 1.2 – 1.8 × 10,000 cells/well containing 100 *µ*l growth medium in a 96-well plate for 24 h previous to performing the MTT assay.

**MTT assay:** MTT (3-[4,5-dimethylthiazole-2-yl]-2,5-diphenyltetrazolium bromide) was used to determine the anti-proliferative activity. Cells were incubated in a moist environment for 24 hours at 37℃. The cells were then treated with various concentrations of the studied substances (0.01, 0.1, 1.0, 10, 50, 100 *µ*M). The media was removed, and the cells were treated for 2 hours with a 5% MTT (200 l) solution/well, allowing the dye to metabolize to colored-insoluble formazan crystals. The remaining solution was discarded and acidified isopropanol was then added for around 30 minutes to dissolve the formazan crystals, which were encased in aluminum foil at room temperature. The absorbance of each well was measured at 450 nm using an Elisa reader. The cell viability was expressed as a percentage of control, and the GI_50_ was calculated using Graph Pad Prism program (Graph Pad software Inc, CA).

**Annexin V-FITC/PI Assay:** The early stages of apoptosis were monitored by Annexin V-FITC (apoptotic cell marker) and PI (necrotic cell marker) double staining. The staining method was used according to the Annexin FITC/PI staining kit (Invitrogen™, Thermo Fisher Scientific Inc.). Briefly, cells were washed in PBS twice and resuspended in 500 *μ*l of PBS plus Annexin V-FITC and PI. For each sample at least 1×104 cells should be analysed using an FACS cytometer.

**In silico Screening:** The designed molecule structures were drawn in PC Model (Version: 10.074, Serena Software, Bloomington) and saved in the .mol format. Later the compounds were energy minimized by using MMFF94 Force filed until the energy difference was 0.001 kJ/mol, and the 3D conformer was saved in .pdb format compound. These 3Dstructures were prepared for docking by using LigPrep tool in Maestro (Schrodinger, LLC, New York, USA) with the standard criteria.The designed molecules were subjected for target prediction by using the Swiss Target Prediction and Pass Online tools. Based on the scores and ratio data from the two servers, potential antitubercular targets like Cyclin-dependent kinase 9 (CDK9)/Cyclin T1 signal transducer and activator of transcription 3 (STAT3) were selected. The X-Ray crystal structures of CDK9/Cyclin T1 (PDB ID: 1BLQ) and STAT3 (PDB ID: 1BG1) were downloaded from RSCB-PDB and was loaded into Maestro.The protein structure preparation was performed as per the default settings. The protein protonation states were maintained at a pH range of 7.0 ± 2.0 and the OPLS3 force-field optimized the geometry with a maximum RMSD of 0.3 Å.Using the Glide software, a grid was created around the chosen binding cavity of the proteins 1BLQ and 1BG1 by picking one atom of the co-crystalized ligand and creating a grid box that measured 16×16×16 Å. The grid file was generated and saved for additional docking investigations using these coordinates and the default settings. Flexible docking was used in the Glide software to determine the potential interactions and affinities between the proposed compounds and the anticipated binding sites of the proteins 1BLQ and 1BG1. To analyze the binding interaction with the proteins 1BLQ and 1BG1, the created receptor grid files were navigated into Glide and the ready ligands were added from workspace using the Extra Precision (XP) docking program with all default parameters left intact.

**4. NMR spectra of representatives compounds (8a-g)**

Figure S1: ^1^H-NMR spectrum of compound **8a** (2-(5-amino-1,3,4-thiadiazol-2-ylthio)-N-(thiazol-2-yl) acetamide) in DMSO-d_6_
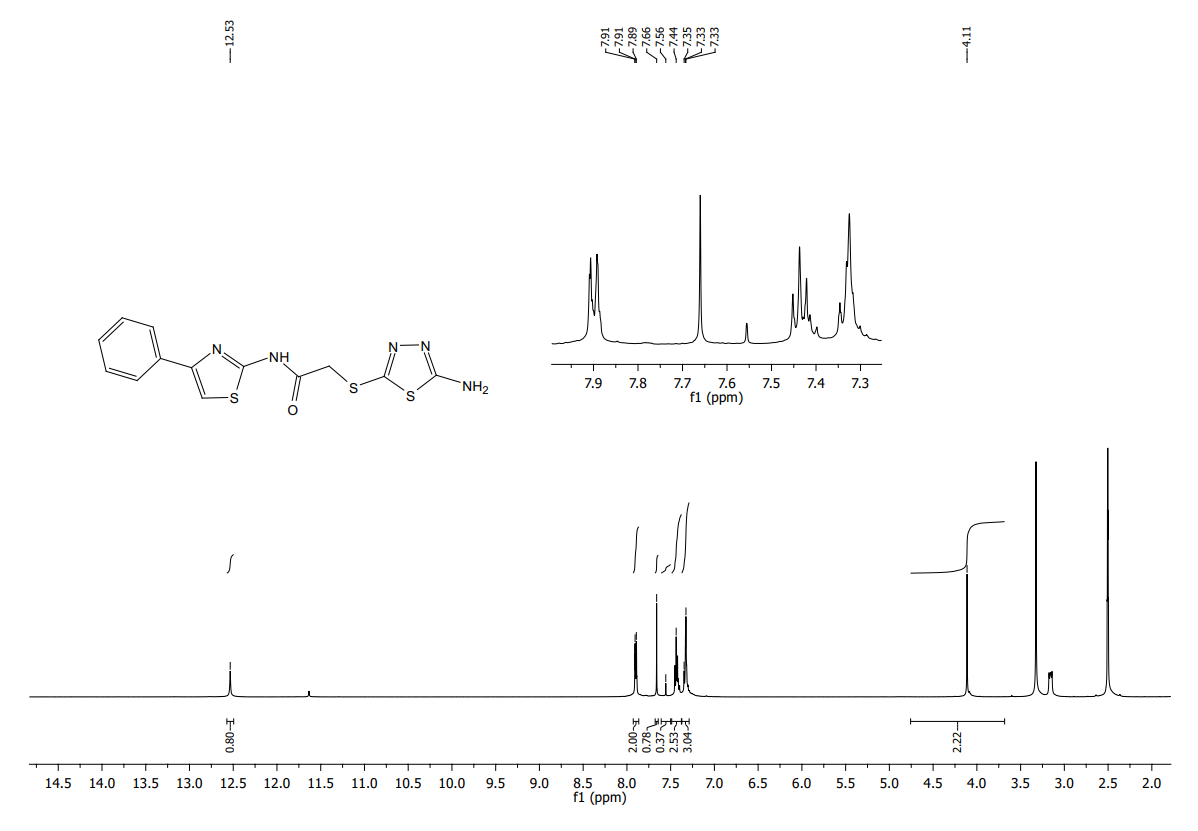


Figure S2: ^13^C-NMR spectrum of compound **8a** (2-(5-amino-1,3,4-thiadiazol-2-ylthio)-N-(thiazol-2-yl) acetamide) in DMSO-d_6_


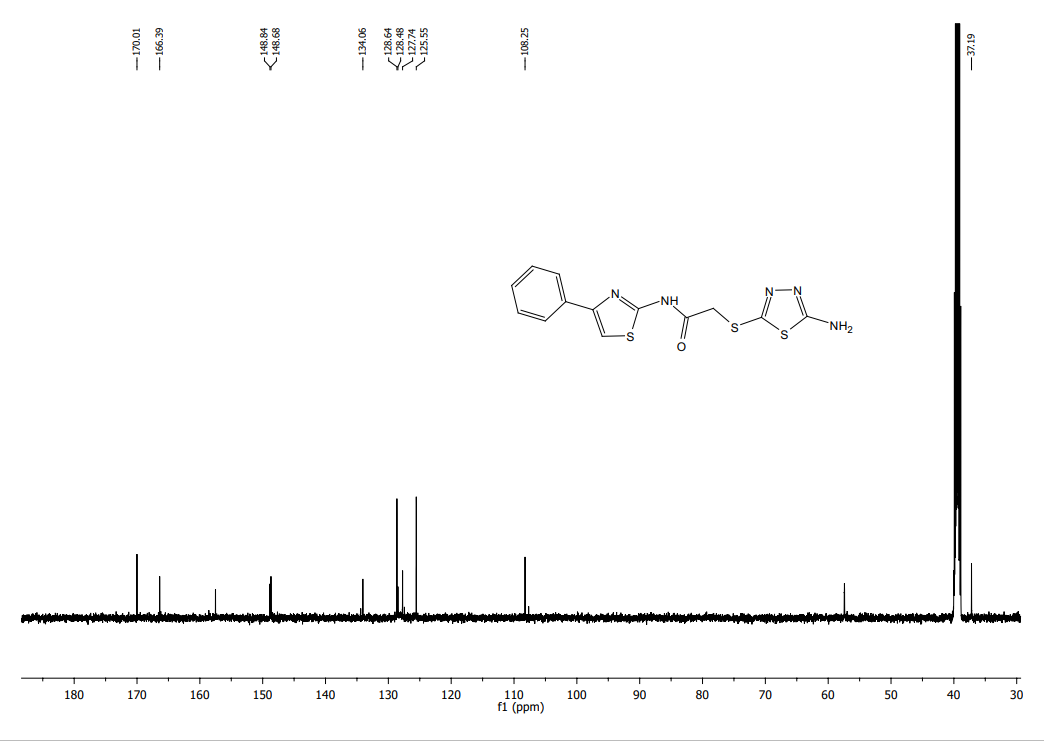


Figure S3: ^1^H-NMR spectrum of compound **8b** (2-(5-amino-1,3,4-thiadiazol-2-ylthio)-N-(4-(4-bromophenyl) thiazol-2-yl) acetamide) in DMSO-d_6_
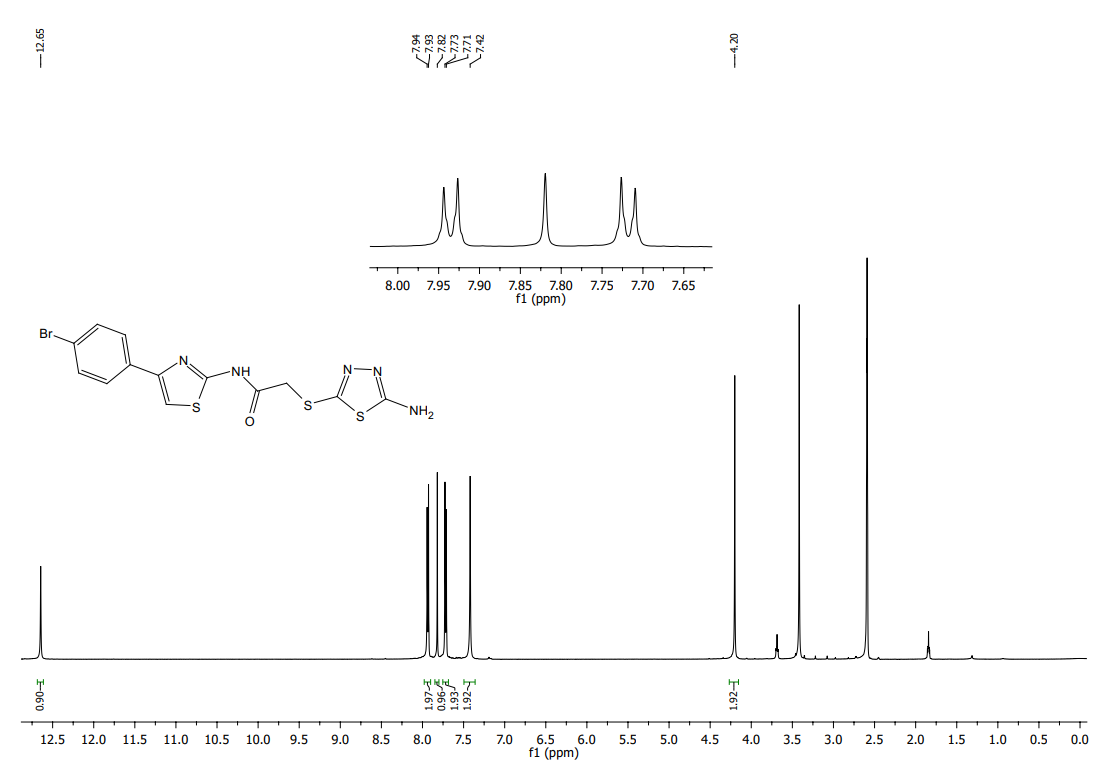


Figure S4: ^13^C-NMR spectrum of compound **8b** (2-(5-amino-1,3,4-thiadiazol-2-ylthio)-N-(4-(4-bromophenyl) thiazol-2-yl) acetamide) in DMSO-d_6_
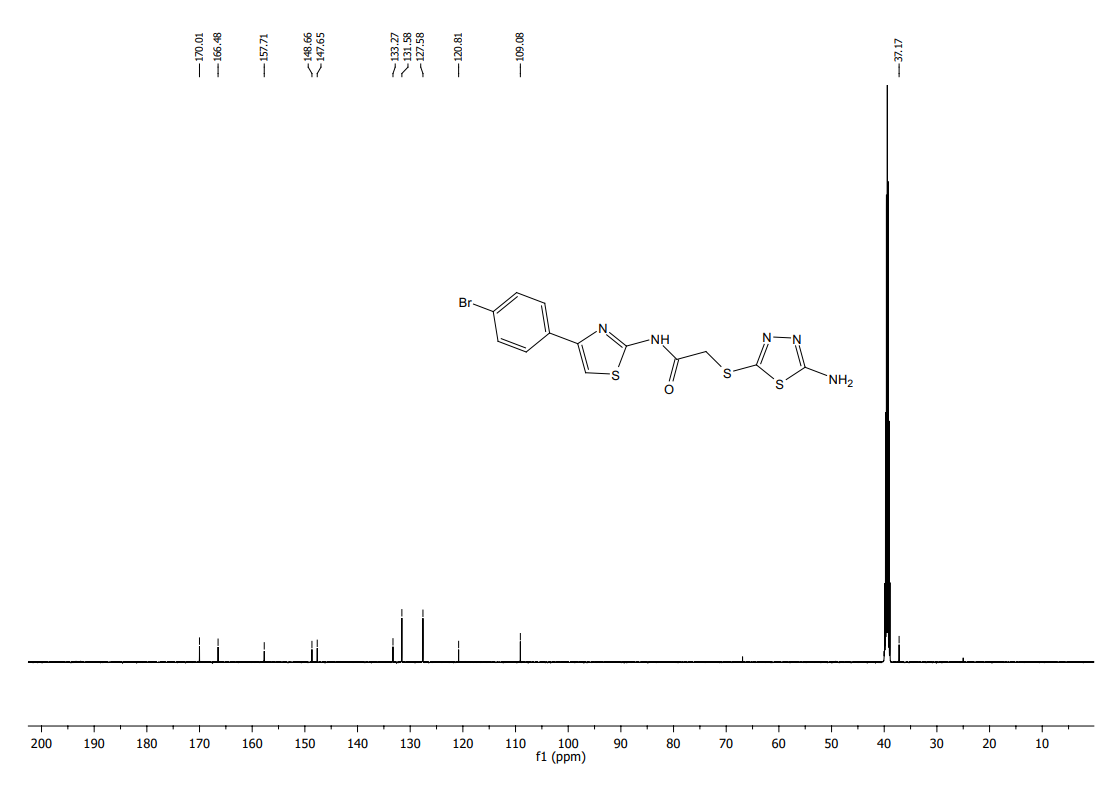


Figure S5: ^1^H-NMR spectrum of compound **8c** (2-(5-amino-1,3,4-thiadiazol-2-ylthio)-N-(4-(4-chlorophenyl) thiazol-2-yl) acetamide) in DMSO-d_6_
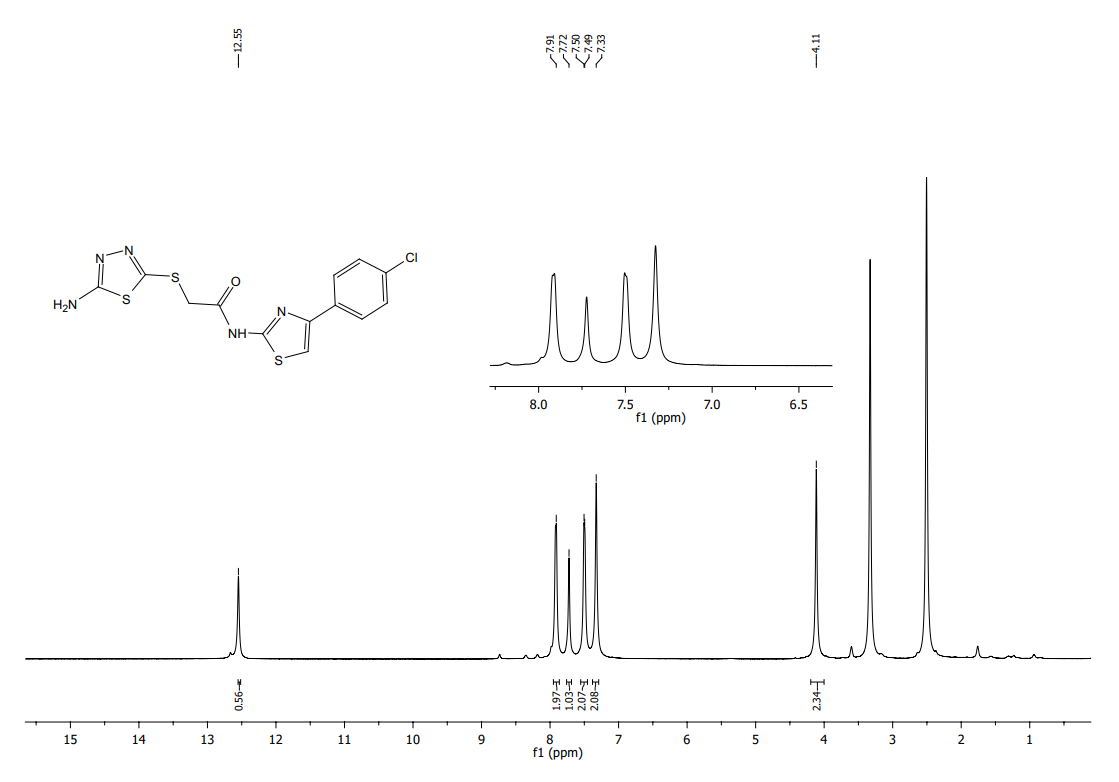


Figure S6: ^13^C-NMR spectrum of compound **8c** (2-(5-amino-1,3,4-thiadiazol-2-ylthio)-N-(4-(4-chlorophenyl) thiazol-2-yl) acetamide) in DMSO-d_6_
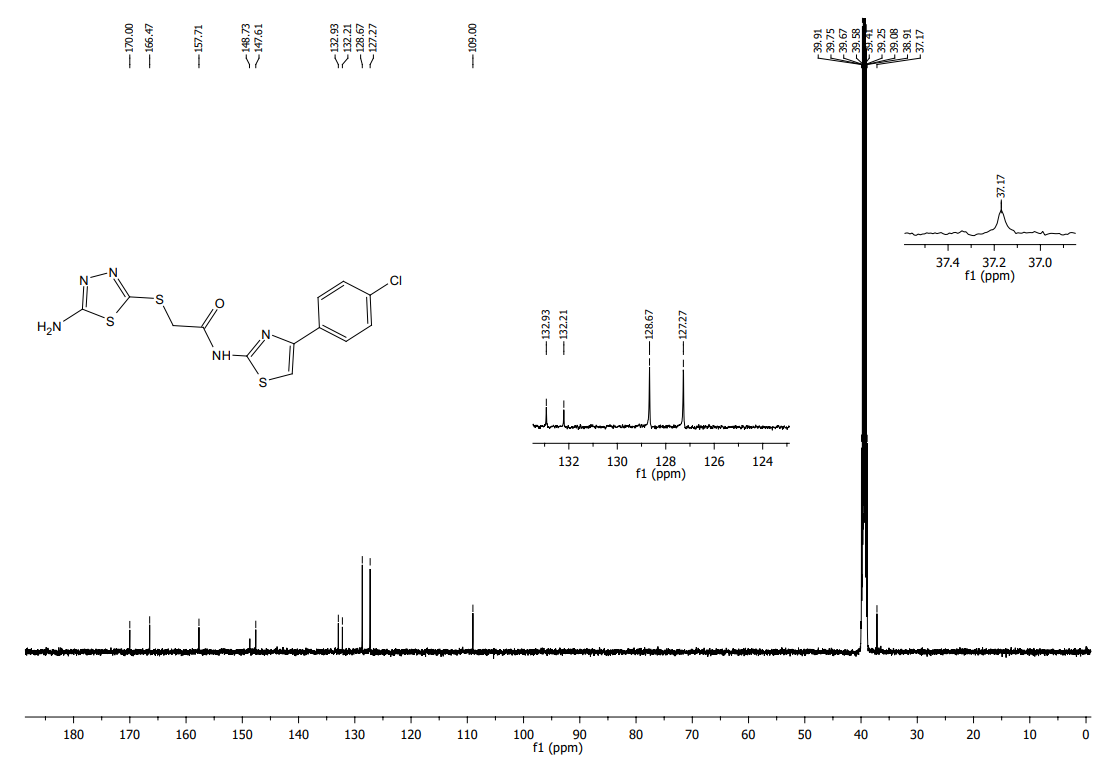


Figure S7: ^1^H-NMR spectrum of compound **8d** (2-(5-amino-1,3,4-thiadiazol-2-ylthio)-N-(4-(4-methoxyphenyl) thiazol-2-yl) acetamide) in DMSO-d_6_
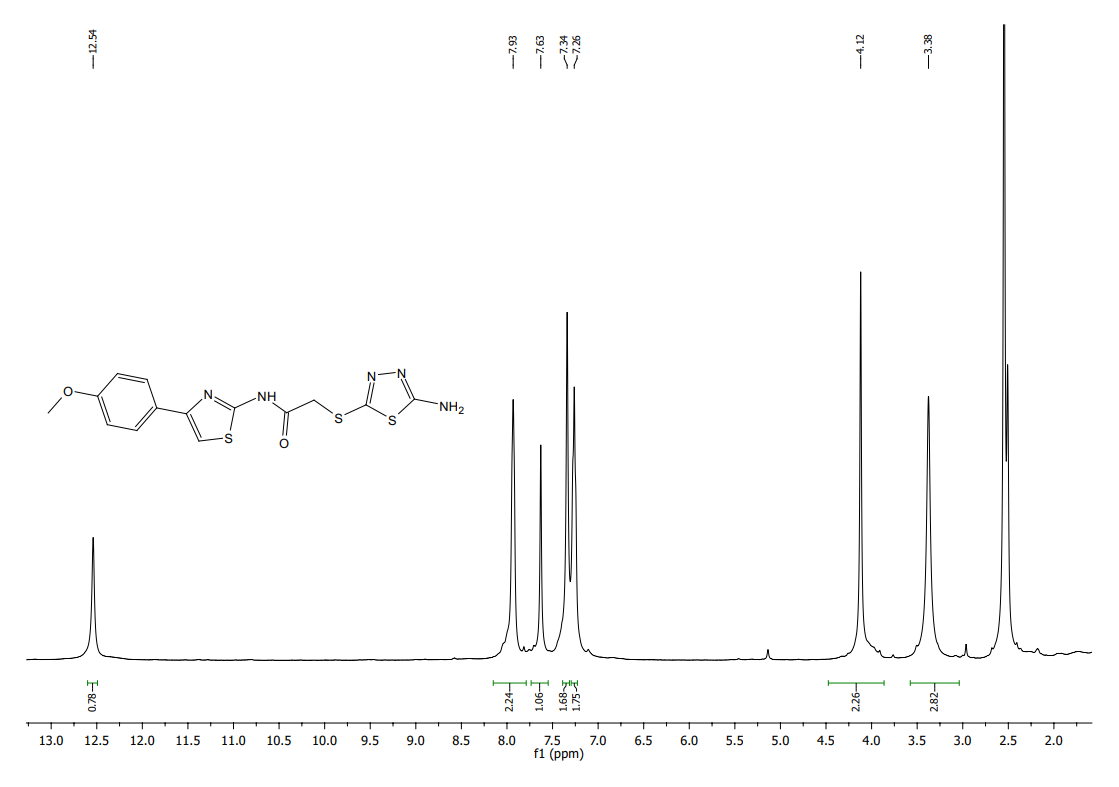


Figure S8: ^13^C-NMR spectrum of compound **8d** (2-(5-amino-1,3,4-thiadiazol-2-ylthio)-N-(4-(4-methoxyphenyl) thiazol-2-yl) acetamide) in DMSO-d_6_


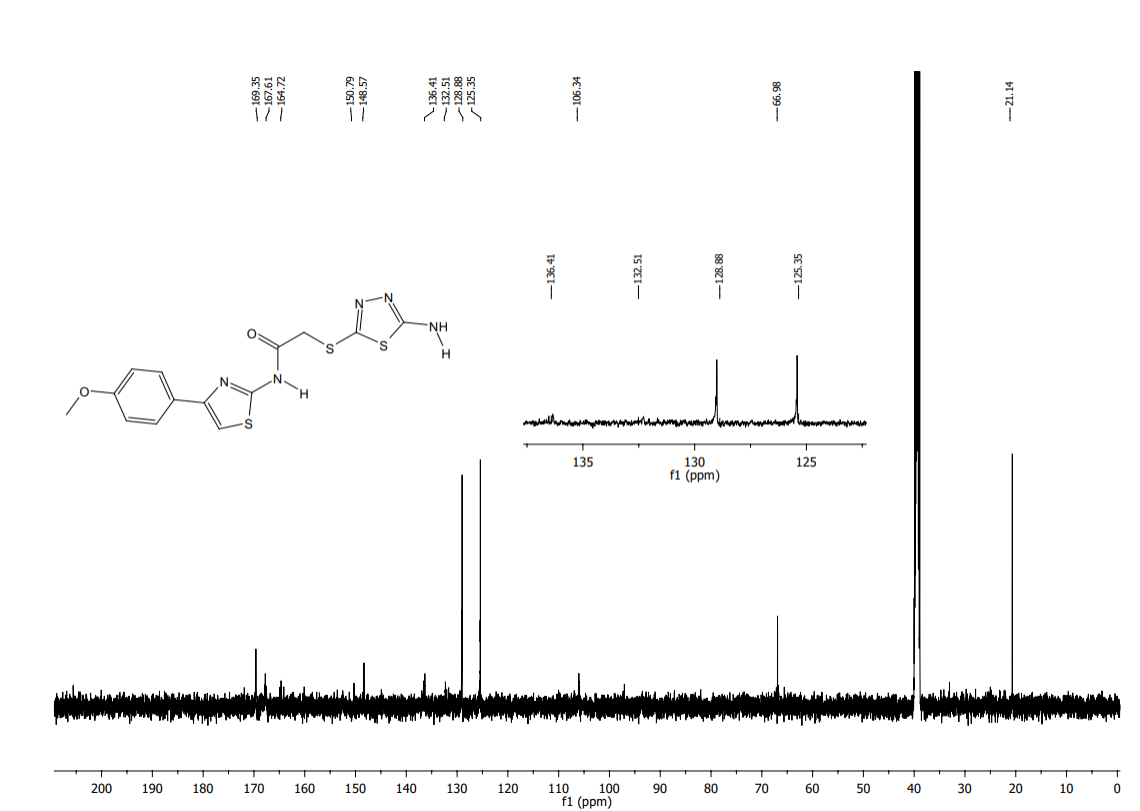


Figure S9: ^1^H-NMR spectrum of compound **8e** (2-(5-amino-1,3,4-thiadiazol-2-ylthio)-N-(4-p-tolylthiazol-2-yl) acetamide) in DMSO-d_6_^
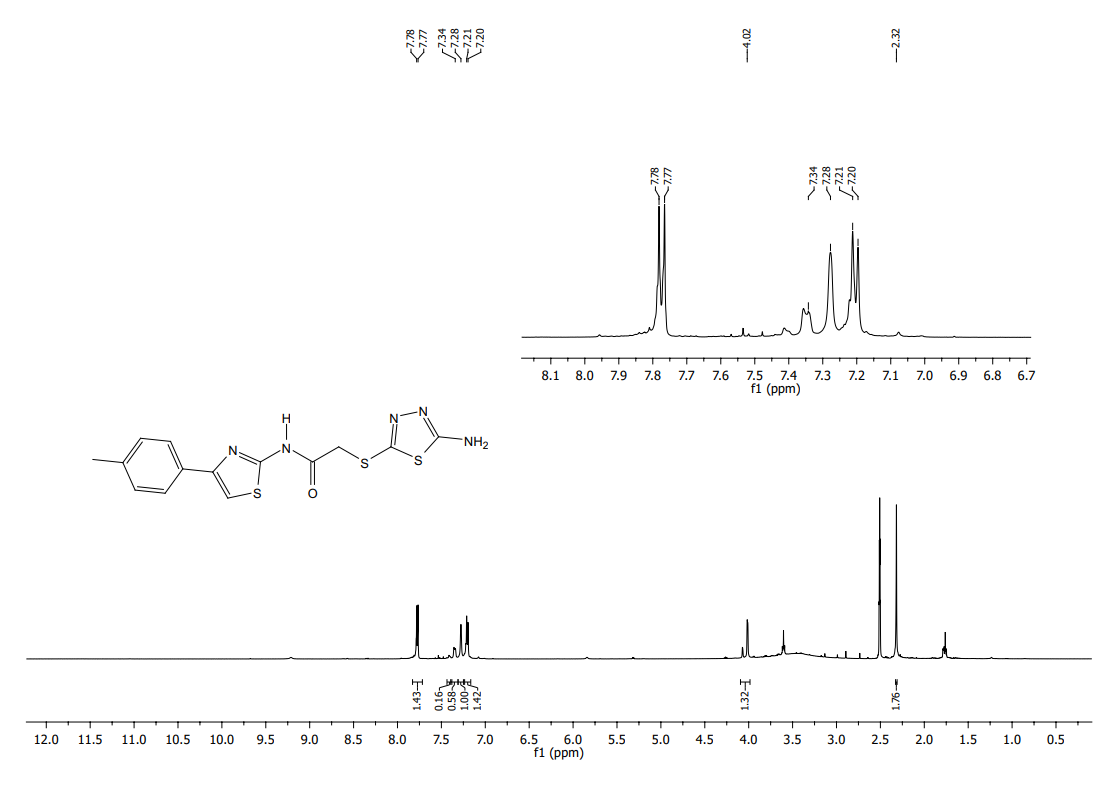
^

Figure S10: ^13^C-NMR spectrum of compound **8e** (2-(5-amino-1,3,4-thiadiazol-2-ylthio)-N-(4-p-tolylthiazol-2-yl) acetamide) in DMSO-d_6_


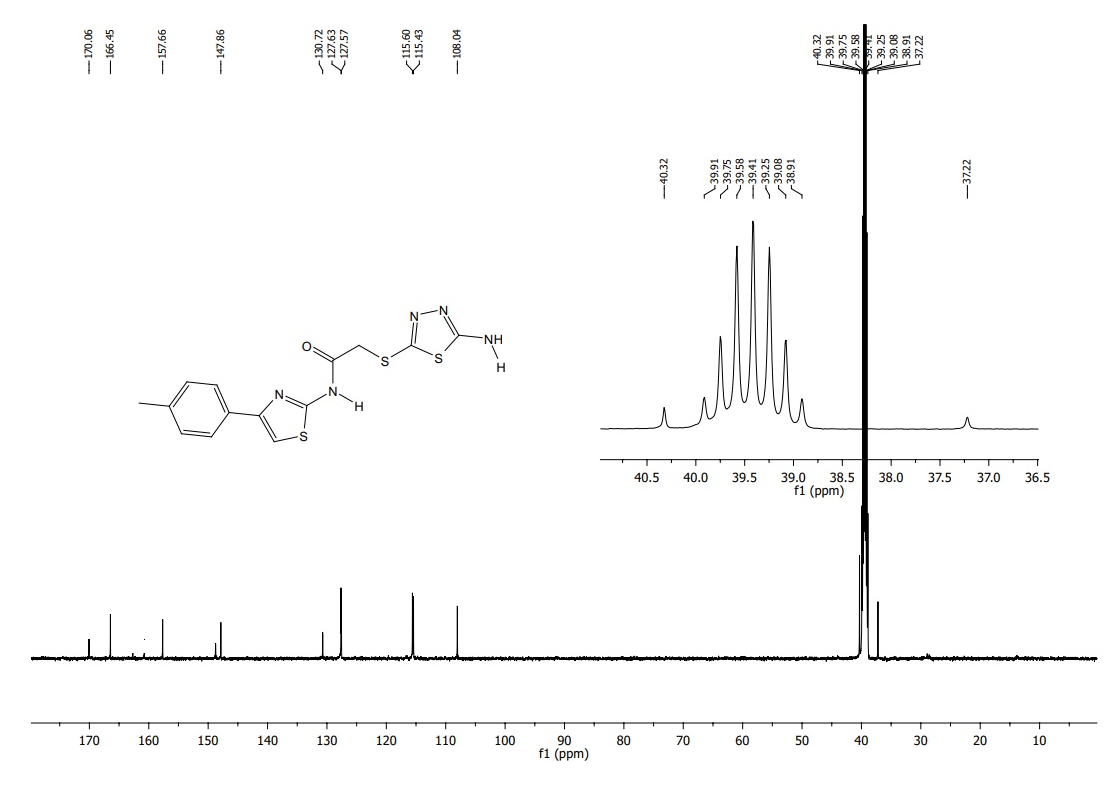


Figure S11: ^1^H-NMR spectrum of compound **8f** (2-(5-amino-1,3,4-thiadiazol-2-ylthio)-N-(4-(4-nitrophenyl) thiazol-2-yl) acetamide) in DMSO-d_6_
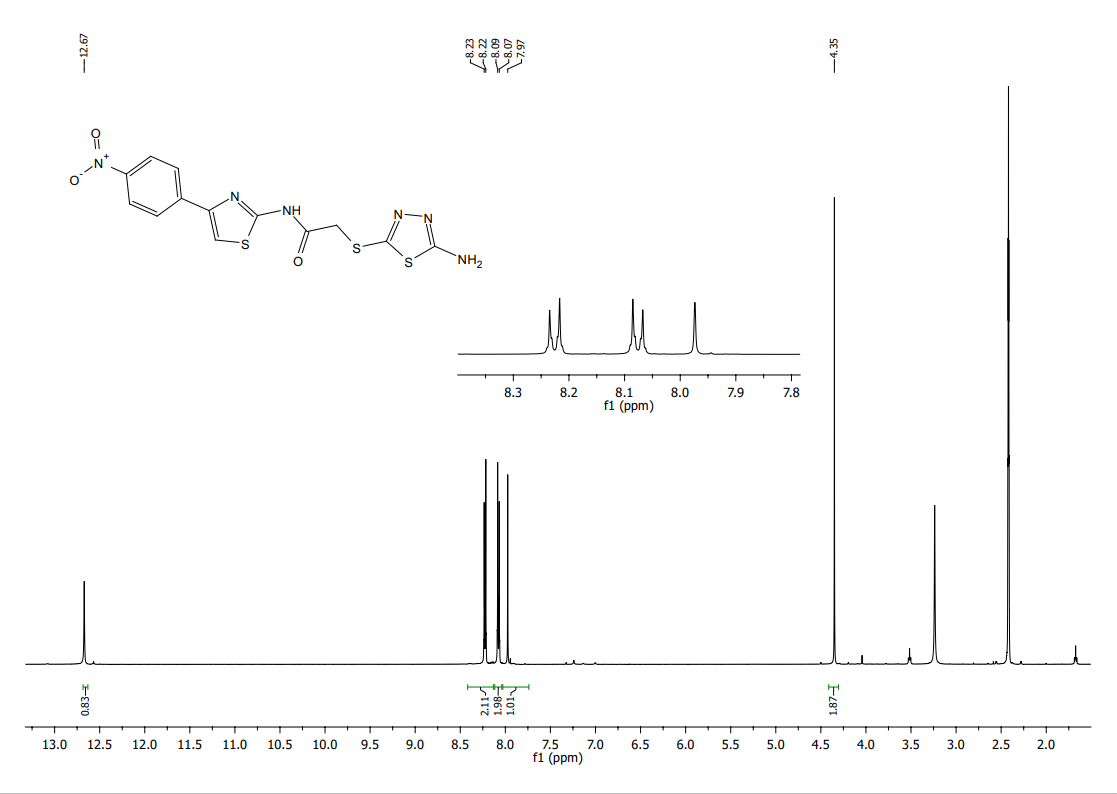


Figure S12: ^13^C-NMR spectrum of compound **8f** (2-(5-amino-1,3,4-thiadiazol-2-ylthio)-N-(4-(4-nitrophenyl) thiazol-2-yl) acetamide) in DMSO-d_6_
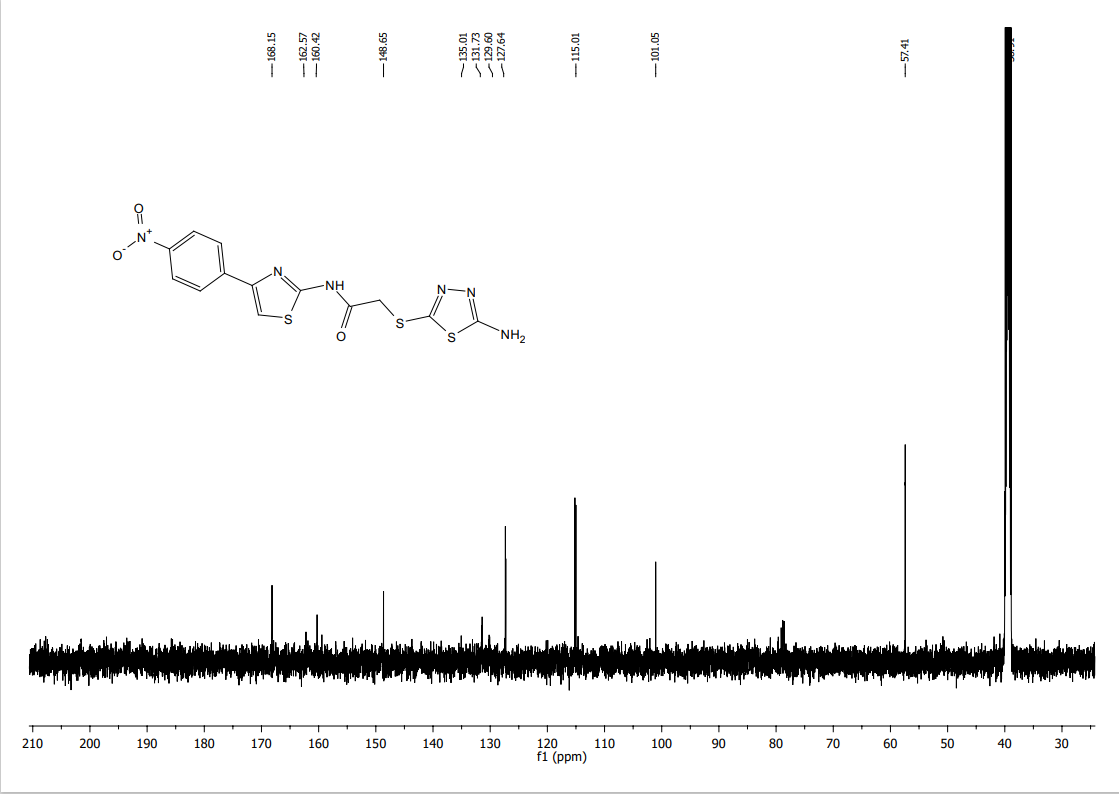


Figure S13: ^1^H-NMR spectrum of compound **8g** (2-(5-amino-1,3,4-thiadiazol-2-ylthio)-N-(4-(4-fluorophenyl) thiazol-2-yl) acetamide) in DMSO-d_6_
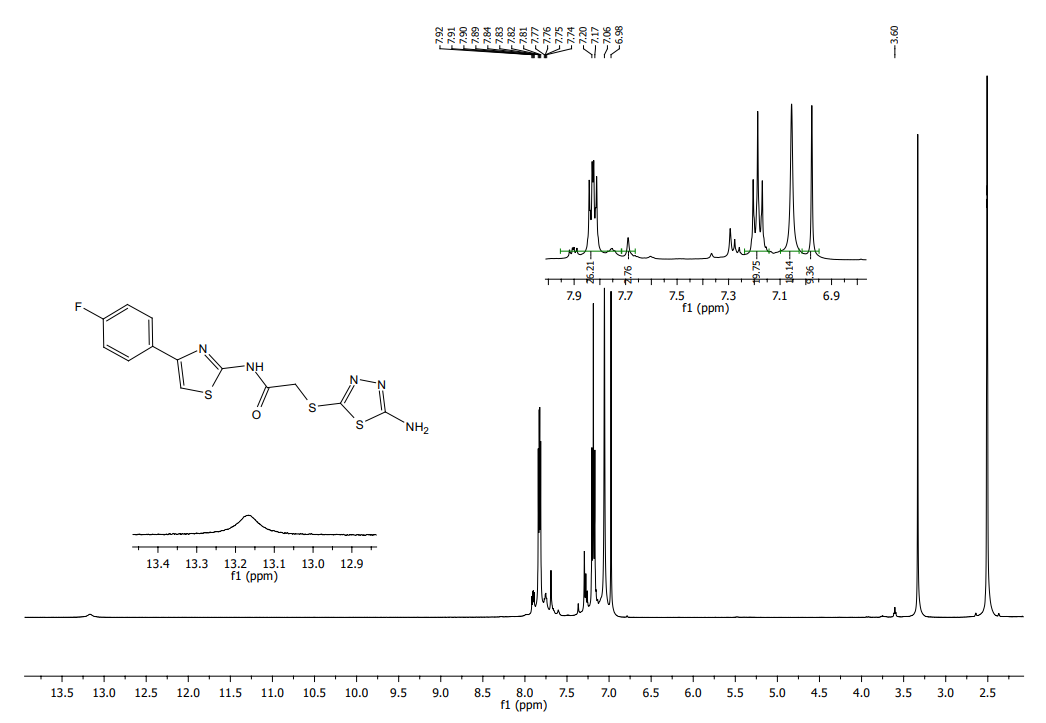


Figure S14: ^13^C-NMR spectrum of compound **8g** (2-(5-amino-1,3,4-thiadiazol-2-ylthio)-N-(4-(4-fluorophenyl) thiazol-2-yl) acetamide) in DMSO-d_6
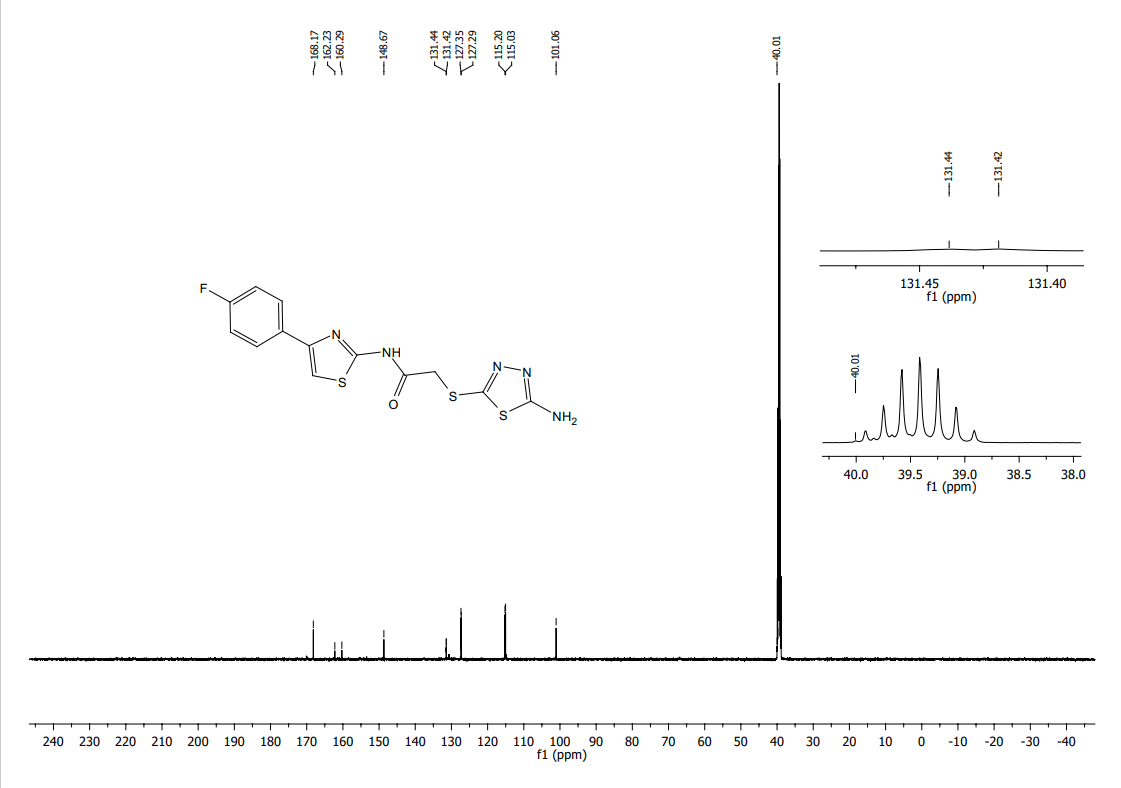
_

**5. Mass spectrum of (8a-g)**

Figure S15: Mass spectrum of **8a**

Figure S16: LC-MS spectrum of **8a**
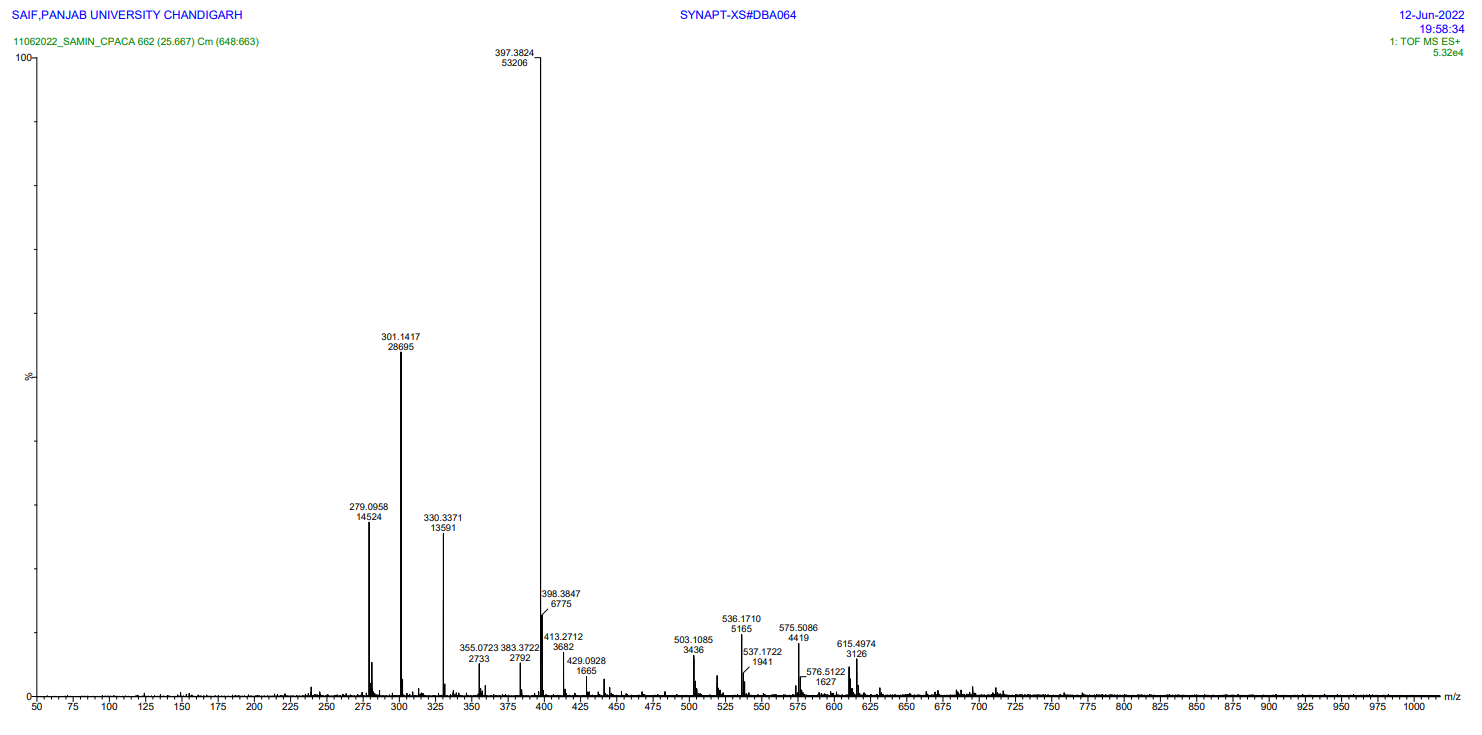


Figure S17: MS spectrum of **8b**
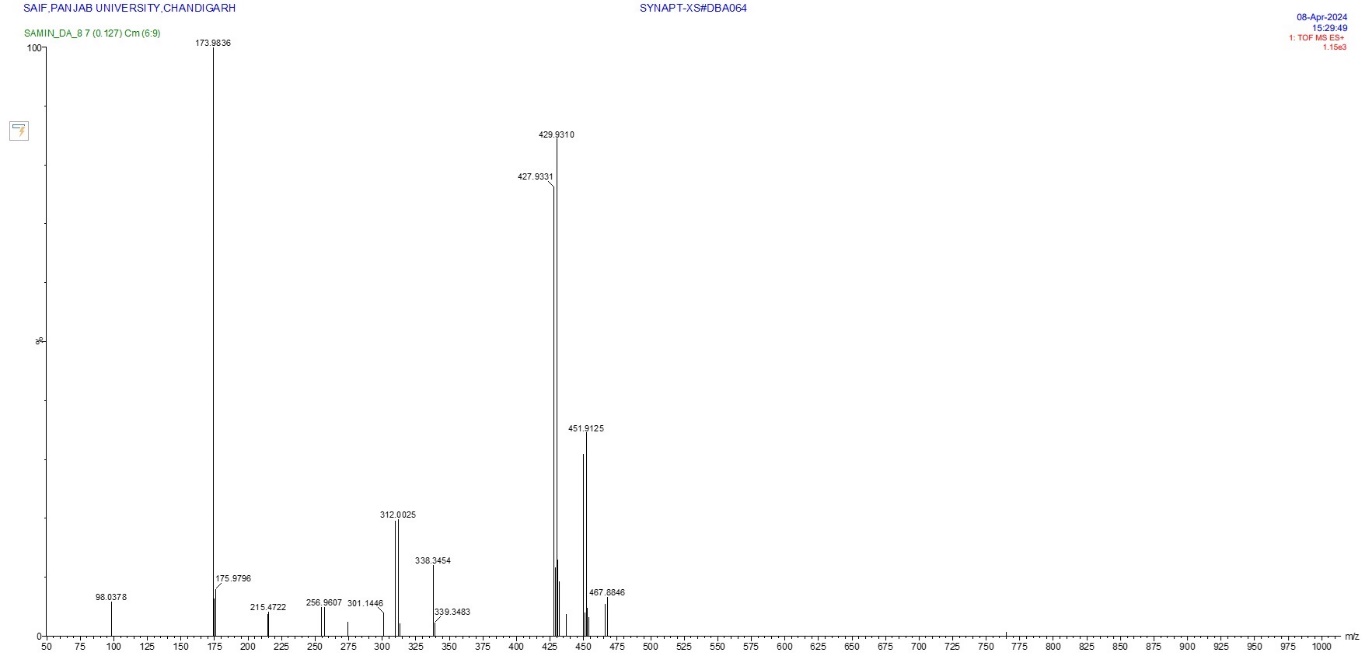


Figure S18:MS spectrum of **8c**
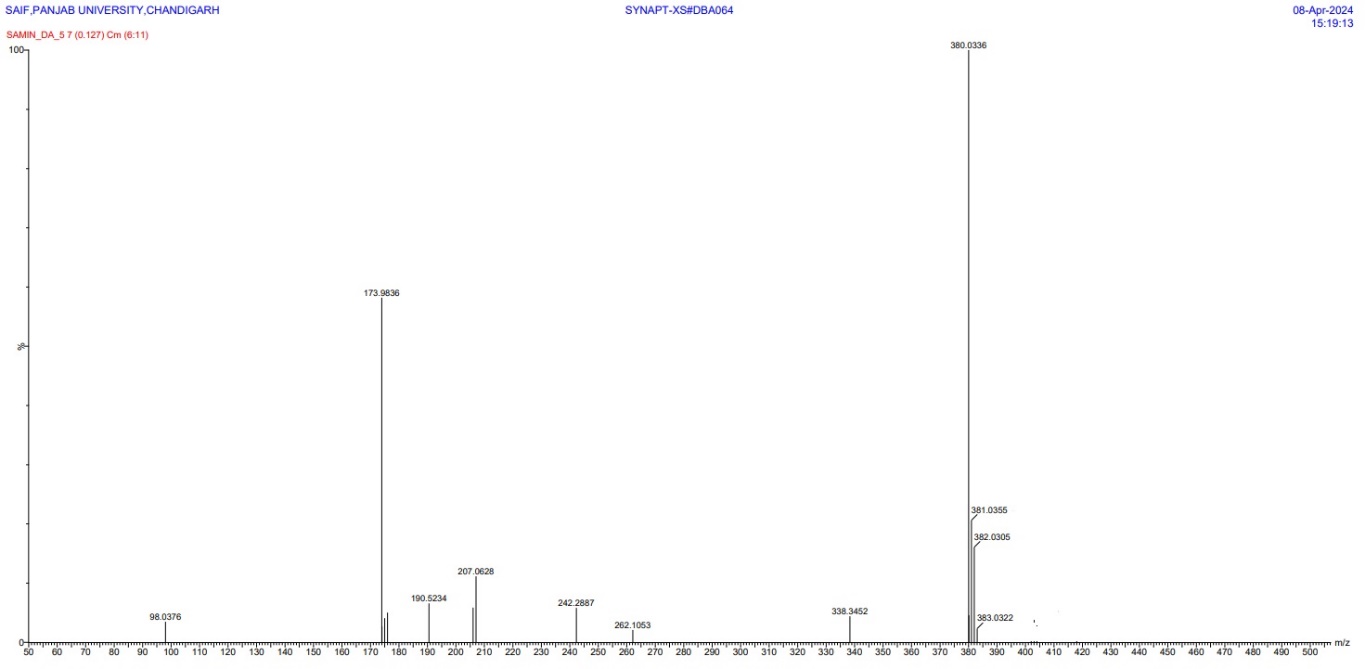


Figure S19: MS spectrum of **8d
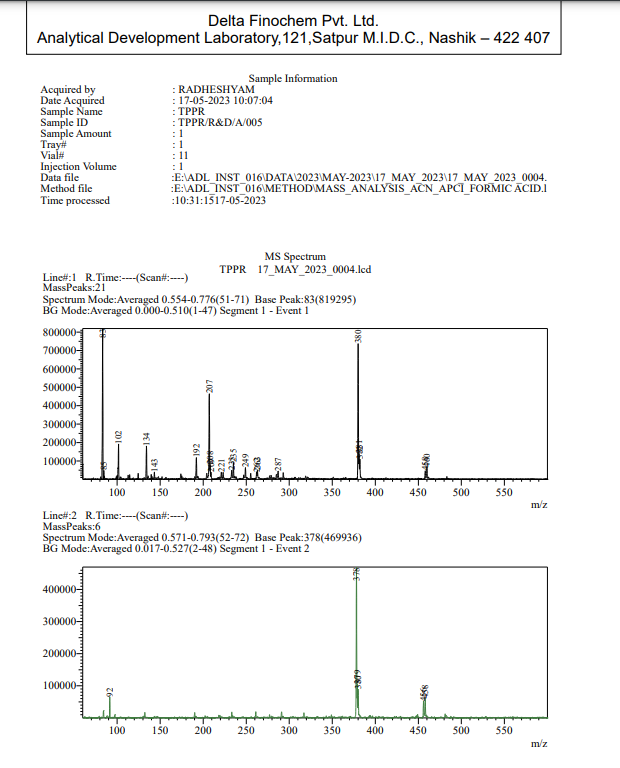
**

Figure S20: LC-MS spectrum of **8e**
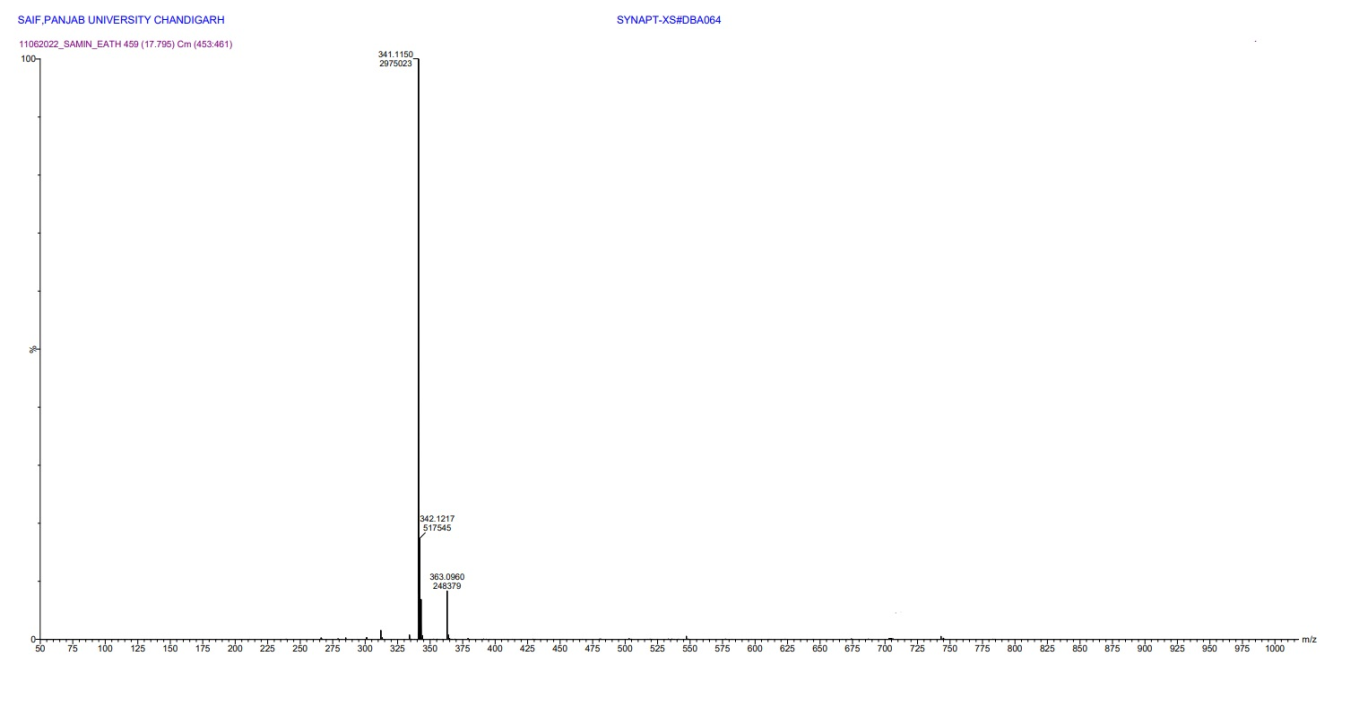


Figure S21: MS spectrum of **8f**

Figure S22: LC-MS spectrum of **8g**
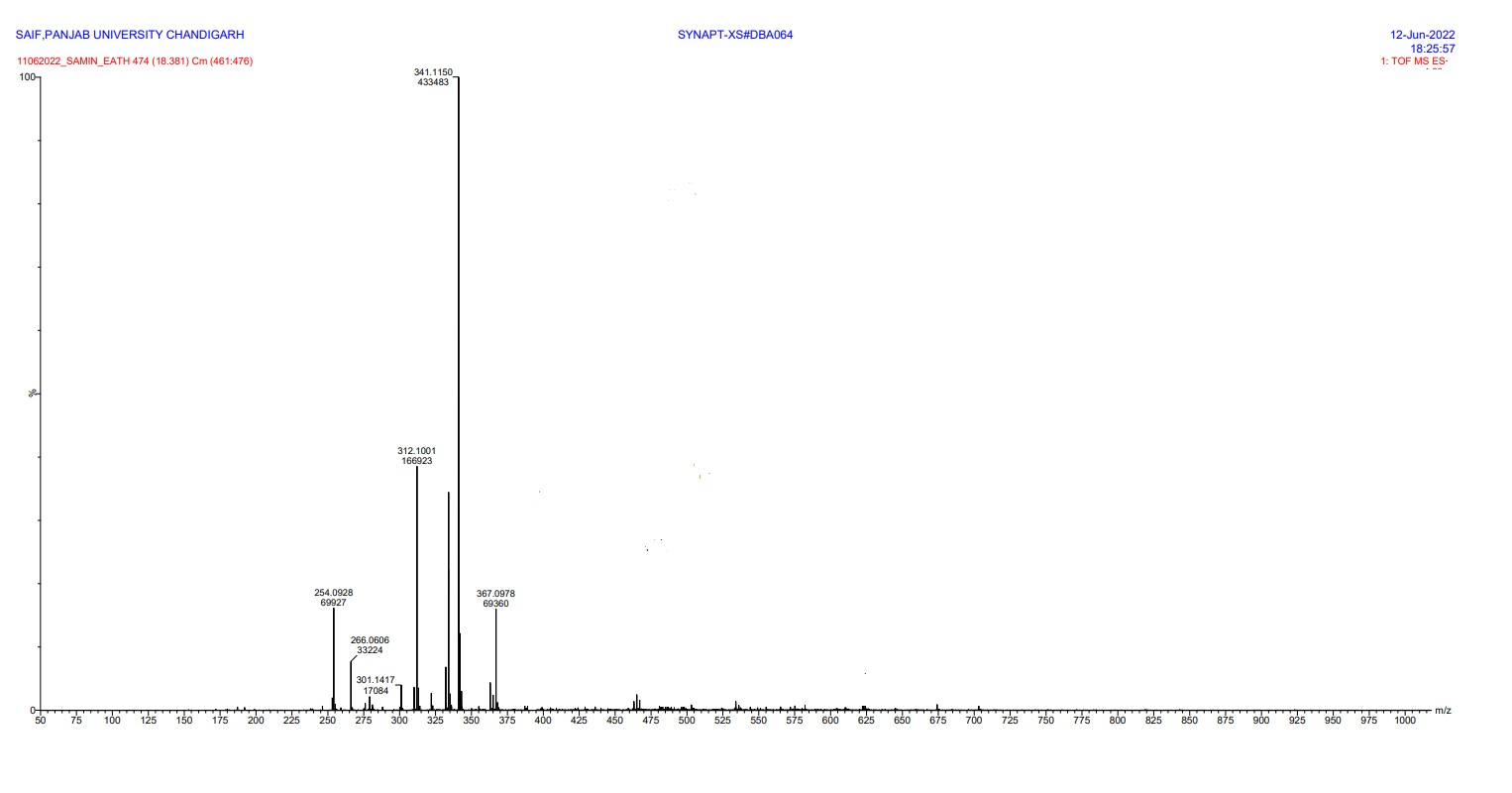


**6. FT-IR spectrum of (8a-8g)**

Figure S23: FTIR spectrum of **8a**
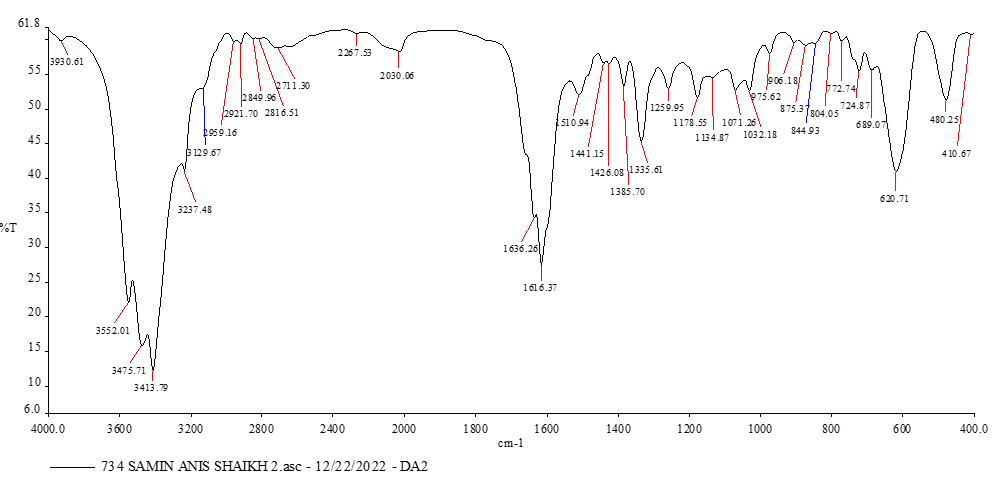


Figure S24: FTIR spectrum of **8b**
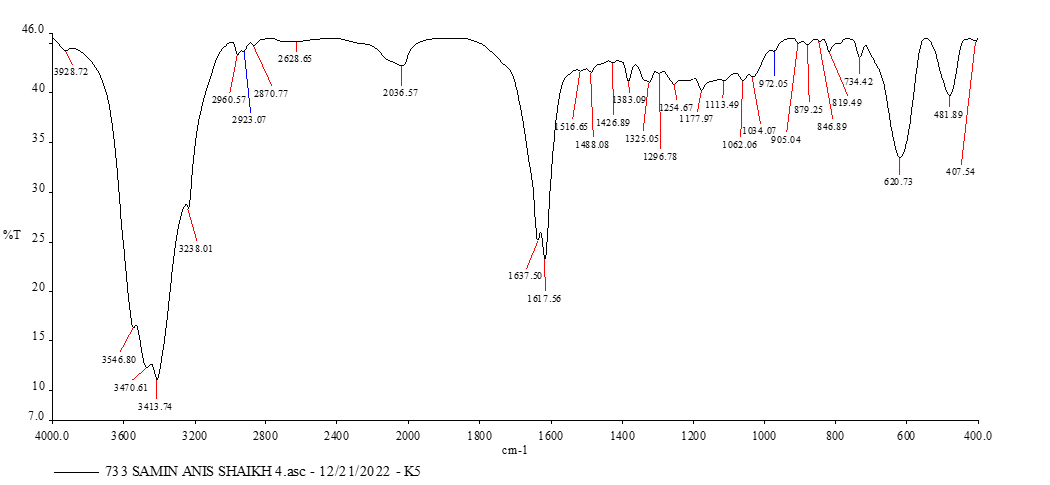


Figure S25: FTIR spectrum of **8c**
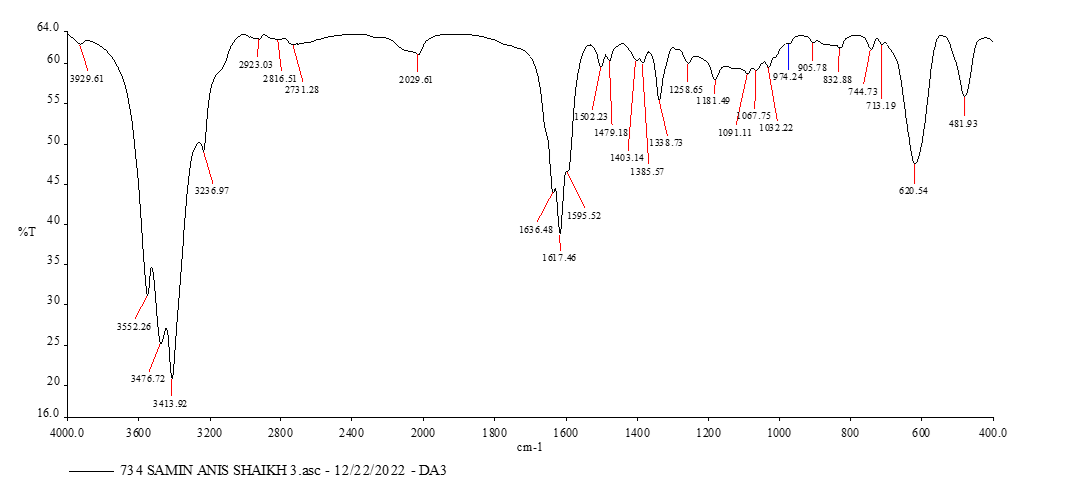


Figure S26: FTIR spectrum of **8d**
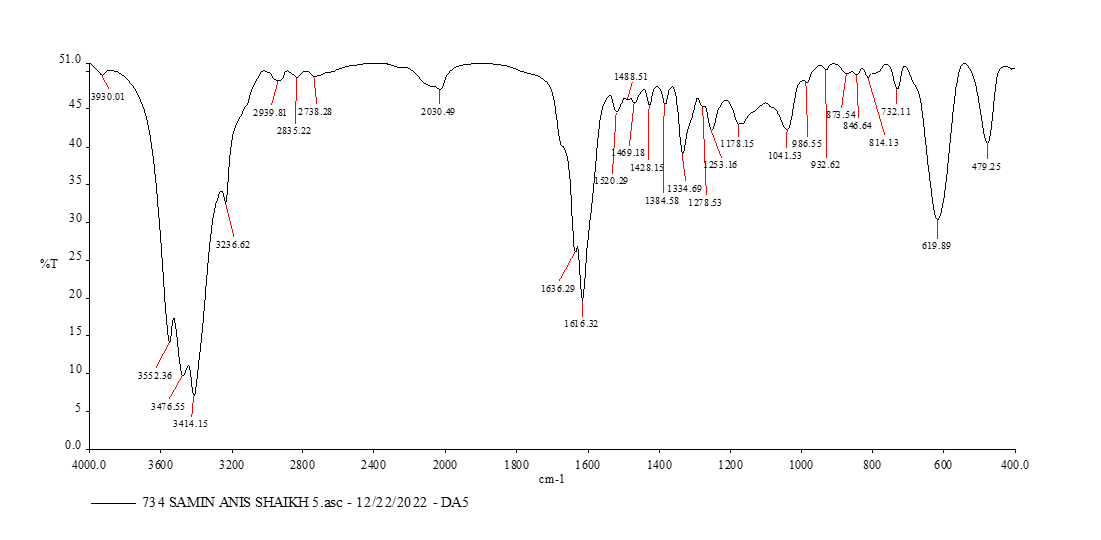


Figure S27: FTIR spectrum of **8e**
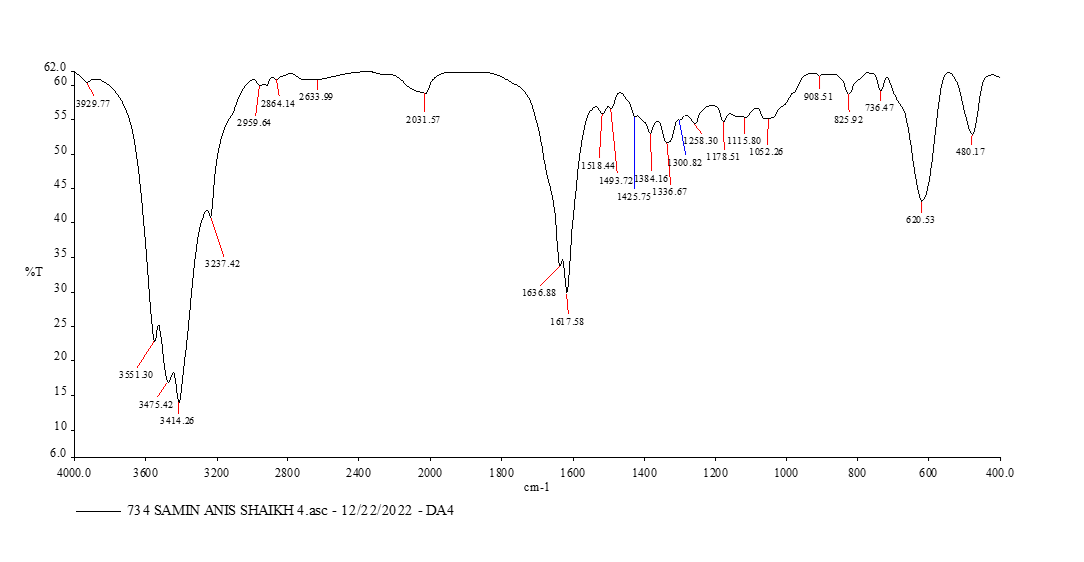


Figure S28: FTIR spectrum of **8f**
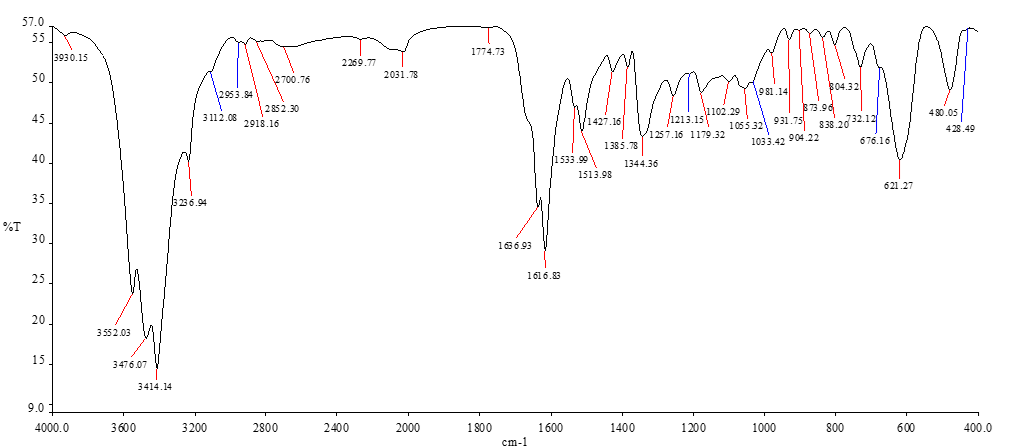


Figure S29: FTIR spectrum of **8g**
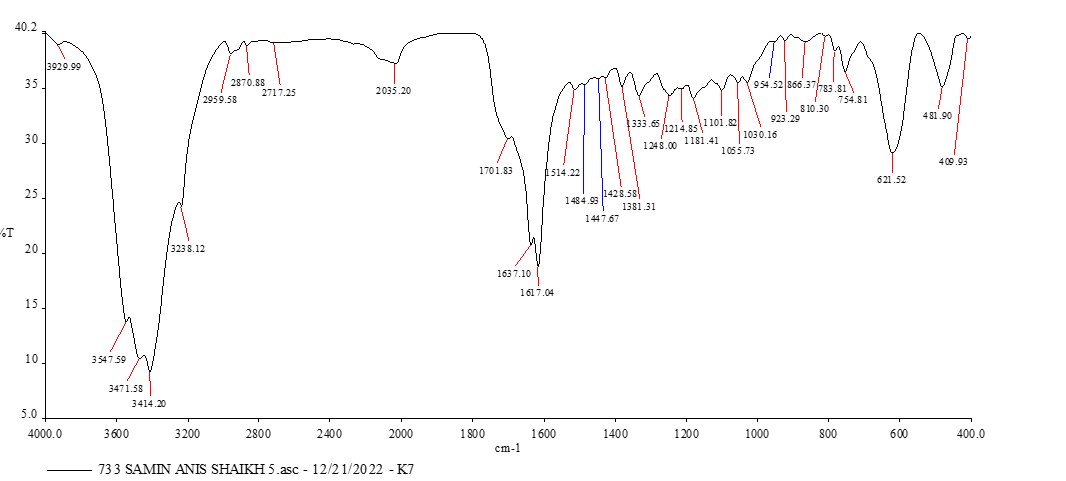

Supplement: Supplementary file 1 — Additional file 1. Additional figures and sections. [file 13065_2024_1196_MOESM1_ESM.docx]
